# Reactions of Dihaloboranes with Electron-Rich 1,4-Bis(trimethylsilyl)-1,4-diaza-2,5-cyclohexadienes

**DOI:** 10.3390/molecules25122875

**Published:** 2020-06-22

**Authors:** Li Ma, Xiaolin Zhang, Wenbo Ming, Shengxin Su, Xiaoyong Chang, Qing Ye

**Affiliations:** Department of Chemistry, Southern University of Science and Technology, Shenzhen 518055, China; 11849423@mail.sustech.edu.cn (L.M.); zhangxl6003@163.com (X.Z.); wenbo.ming@hotmail.com (W.M.); 11510019@mail.sustech.edu.cn (S.S.); changxy@sustech.edu.cn (X.C.)

**Keywords:** 1,4-bis(trimethylsilyl)-1,4-diaza-2,5-cyclohexadienes, salt-free reduction, rotational barrier, B=N bond

## Abstract

The reactions of electron-rich organosilicon compounds 1,4-bis(trimethylsilyl)-1,4-diaza-2,5-cyclohexadiene (**1**), 2,3,5,6-tetramethyl-1,4-bis(trimethylsilyl)-1,4-diaza-2,5-cyclohexadiene (**2**), and 1,1′-bis(trimethylsilyl)-1,1′-dihydro-4,4′-bipyridine (**12**) with *B*-amino and *B*-aryl dihaloboranes afforded a series of novel B=N-bond-containing compounds **3**–**11** and **13**. The B=N rotational barriers of **7** (>71.56 kJ/mol), **10** (58.79 kJ/mol), and **13** (58.65 kJ/mol) were determined by variable-temperature ^1^H-NMR spectroscopy, thus reflecting different degrees of B=N double bond character in the corresponding compounds. In addition, ring external olefin isomers **11** were obtained by a reaction between **2** and DurBBr_2_. All obtained B=N-containing products were characterized by multinuclear NMR spectroscopy. Compounds **5**, **9**, **10a**, **11**, and **13a** were also characterized by single-crystal X-ray diffraction analysis.

## 1. Introduction

Low-valent boron compounds are a class of highly reactive species that have been the focus of intense research because of their unique electronic properties [1,2] as well as their diverse and fascinating reactivity patterns such as inert bond activation [3,4], cycloaddition reaction [5,6,7], and small molecule activation [8]. The progress in this research area is highlighted by the very recent results in terms of borylene-mediated N_2_ activation [9] and N_2_ coupling [10]. Nonetheless, the synthetic approach to low-valent boron species is severely limited [11,12,13]. Almost all of the reported synthetic strategies require a strong metallic reducing agent (e.g., Li, K, Na, KC_8_) [3,4,14,15,16,17,18,19,20,21,22,23], harsh reaction conditions, and a strict moisture- and oxygen-free atmosphere. Therefore, the exploration of metal-free reductants to access low-valent boron species is highly desirable [24,25,26].

**Scheme 1:**
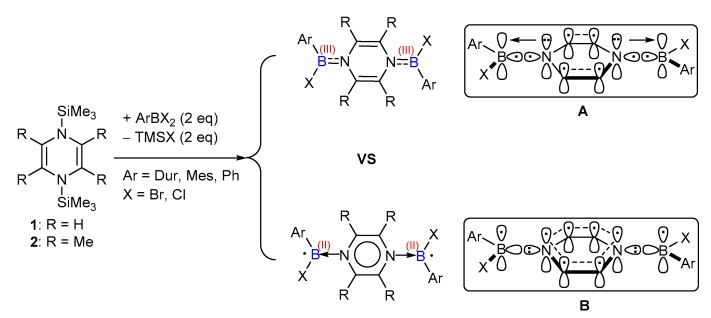
Proposed products from the reactions of ArBX_2_ with **1** and **2**, and two possible bonding modes **A** and **B** between the central C_4_N_2_ ring and the boron centers.

Mashima et al. reported a class of electron-rich organosilicon compounds **1**, **2**, and **12**, which can serve as versatile reducing reagents for the group 4–6 metal chloride complexes. The corresponding low-valent metal species were prepared in a salt-free manner [27,28,29,30,31,32,33]. The reducing power mainly derives from the aromatization of the central 1,4-diaza-2,5-cyclohexadiene ring. Deeply inspired by the advantage of the salt-free reduction protocol and easy workup, we decided to examine the ability of the organosilicon compounds **1**, **2**, and **12** for the reduction of trivalent dihaloboranes. Based on the published results, the disubstituted compounds ArXB(N_2_C_4_R_4_)BXAr are proposed as the reduction products. We hypothesized two possible bonding modes (i.e., **A** and **B** in Scheme 1) between the C_4_N_2_ ring and the boron atoms. In the first manner, B–N is bound by an electron-precise σ bond (**A**, Scheme 1) and an additional N―B dative π bond. In the second manner, two nitrogen atoms each provide a π-electron for 6π-aromatization, while the remaining two valence electrons form a lone pair on each N atom, donating to the empty *sp^2^*-hybridized orbital of boron, thus leading to the divalent boron radical centers (**B**, Scheme 1).

## 2. Results and Discussion

**Scheme 2:**
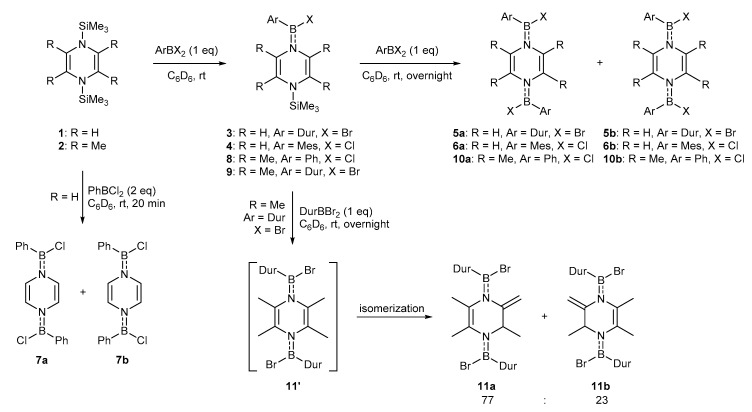
Synthesis of **3**–**11**.

First, we examined compound **1** for its ability to reduce ArBX_2_. The results are summarized in Scheme 2. Compound **1** [34] and ArBX_2_ (Ar = 2,3,5,6-tetramethylphenyl (Dur), 2,4,6-trimethylphenyl (Mes)) [35] were prepared according to the literature. The reaction of **1** with an equimolar amount of DurBBr_2_ and MesBCl_2_ at ambient temperature afforded the expected monosubstituted products **3** and **4**, respectively. Adding the second equiv. of dihaloboranes to the reaction mixture led to the disubstituted products **5** and **6**. In stark contrast, the reaction of **1** with an equimolar amount of the less sterically demanding PhBCl_2_ caused precipitation, which is insoluble in all ordinary solvents. This is most likely due to the polymerization of PhClBC_4_N_2_H_4_SiMe_3_ by chlorosilane elimination. Hence, the stepwise synthetic protocol is unsuitable for the synthesis of **7**. Instead, **1** was directly treated with 2 equiv. of PhBCl_2_ at room temperature (RT), affording **7** in an acceptable yield (48%). Hence, the reaction of the monosubstituted intermediate (i.e., PhClBC_4_N_2_H_4_SiMe_3_) with PhBCl_2_ should be much faster than the self-polymerization process. Compounds **3**–**7** were confirmed by NMR spectroscopic (Figures S1–S15) and HRMS studies. Furthermore, the multinuclear NMR spectroscopic study revealed that the isolated **5**–**7** all consist of ca. 1:1 *cis-trans* isomers in the solution phase at ambient temperature due to the nonrotatable B=N double bond (see Electronic Supporting Information (ESI)).

**Figure 1:**
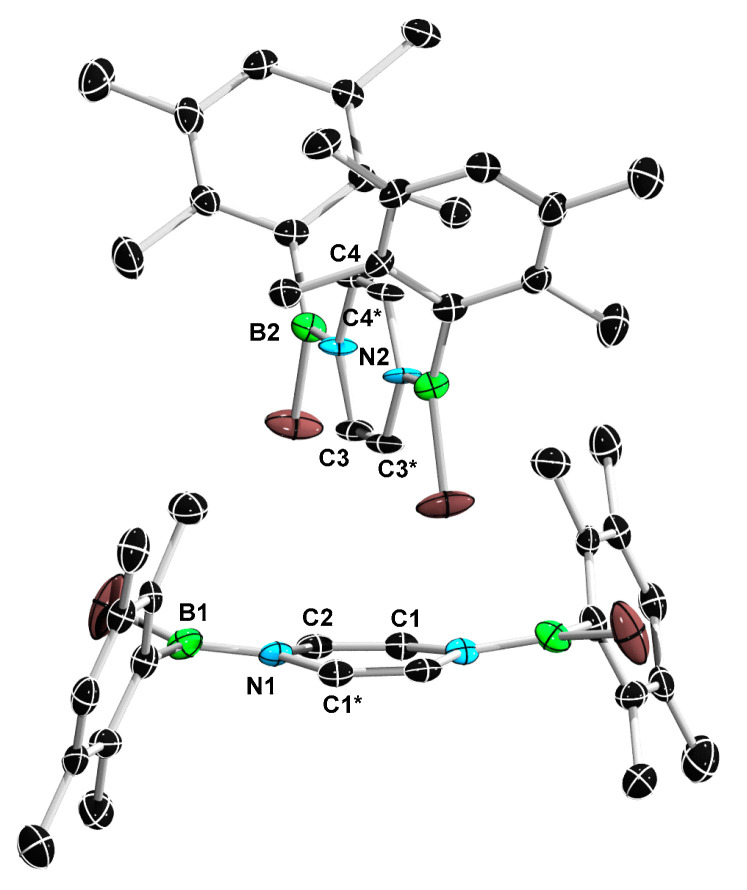
Molecular structures of **5a** (bottom) and **5b** (top) in the solid state (ellipsoids set at 50% probability). Hydrogen atoms are omitted for clarity. Selected bond lengths (Å) and angles (°) for **5a**: B1–N1 1.423(8), N1–C2 1.385(7), C1–C2 1.311(9), C1–C2–N1 123.1(5), C2–N1–C1* 113.9(4); for **5b**: B2–N2 1.400(8), N2–C4 1.411(6), N2–C3 1.415(7), C4–C4* 1.328(8), C3–C3* 1.326(8), C4*–C4–N2 123.7(4), C4–N2–C3 112.6(4), N2–C3–C3* 123.7(5).

Suitable single crystals of **5** for X-ray diffraction analysis were obtained by slow evaporation of a saturated hexane solution. Two isomers, **5a** and **5b**, co-crystallized in the unit cell. The result is depicted in Figure 1 and Figure S37. The central C_4_N_2_ ring is nearly planar. The endocyclic N1–C2 (1.385(7) Å), N2–C3 (1.415(7) Å), C1–C2 (1.311(9) Å), and C3–C3* (1.326(8) Å) distances lie in the expected range for N–C single bonds and C=C double bonds. The bond lengths of B1–N1 (1.423(8) Å) and B2–N2 (1.400(8) Å) are shorter than that of a B–N single bond, which is indicative of a significant B=N double bond character. All these geometric parameters suggest the bonding mode **A** in Scheme 1. Therefore, both boron centers adopt a formal oxidation state of +3.

Compound **2**, which features a less-negative redox potential (+0.10 V) with respect to **1** (−0.24 V), was further examined to reduce PhBCl_2_ and DurBBr_2_ (Scheme 2). Differing from the aforementioned reactions with **1**, both monosubstituted products **8** and **9** could be prepared upon a 1:1 ratio reaction of **2** with PhBCl_2_ and DurBBr_2_, respectively. Upon the reaction of **2** with two equiv. of PhBCl_2_ at RT, the disubstituted compounds **10a** and **10b** were obtained as 1:1 *cis-trans* isomers. Surprisingly, treatment of **2** with two equiv. of DurBBr_2_ at ambient temperature led to the formation of **11**, which can be regarded as the product from an isomerization of **11′** [36,37]. Compounds **8**–**11** were confirmed by NMR spectroscopic (Figures S16–S28) and HRMS studies. There were two sets (intensity ratio of ca. 1:0.3) of ^1^H signals between 4 and 6 ppm, each consisting of three multiplets with the integration ratio of 1:1:1, which can be assigned to the migrated H and two remaining olefinic protons. These are the most characteristic signs for the formation of the isomerized product. After assigning each peak (with the help of the NOE spectrum, see the ESI Figure S26 for more details), we could determine that the ratio of isomers **11a** and **11b** was 77:23. Furthermore, the isomerization was also observed upon the treatment of the isolated **9** with an equimolar amount of DurBBr_2_ at RT.

**Figure 2:**
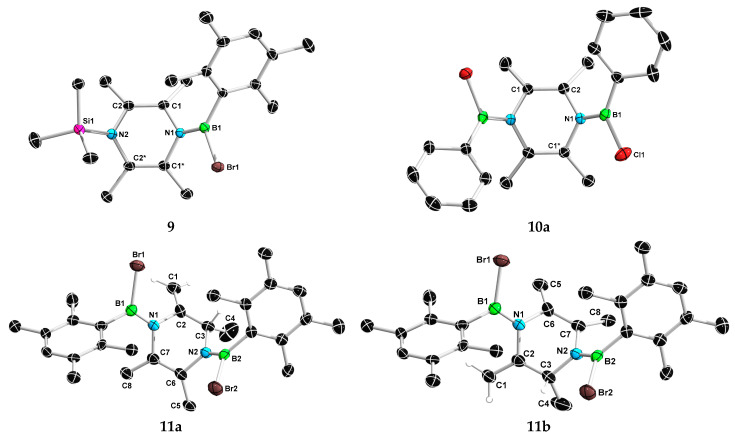
Molecular structures of **9**, **10a**, and **11** in the solid state (ellipsoids set at 50% probability). Hydrogen atoms, except for the C(sp^3^)–H and the olefinic H in **11a** and **11b**, are omitted for clarity. Selected bond lengths (Å) and angles (°) for **9**: B1–N1 1.376(7), N1–C1 1.451(6), C1–C2 1.343(7), N2–C2 1.434(6), N2–Si1 1.759(5), C2–C1–N1 115.3(4), C1–N1–C1* 111.1(4); for **10a**: B1–N1 1.401(2), N1–C2 1.4494(17), C1–C2 1.328(2), N1–C1* 1.4513(18), C1–C2–N1 116.25(12), C2–N1–C1* 110.63(11).

The structures of **9**, **10a**, **11a**, and **11b** were confirmed by single-crystal X-ray diffraction analysis (Figure 2 and Figures S38–S40). All four compounds adopt a boat conformation, which could be explained by the small energy difference between the planar and nonplanar geometry of the C_4_N_2_ ring, and the steric congestion between the central exocyclic methyl groups and the bulky boron substituents. Bond lengths (Å) of **9** (B1–N1 1.376(7), N1–C1 1.451(6), C1–C2 1.343(7), N2–C2 1.434(6), N2–Si1 1.759(5)), **10** (B1–N1 1.401(2), N1–C2 1.4494(17), C1–C2 1.328(2), N1–C1* 1.4513(18)) are all as expected. The overall structures of **11a** and **11b** resemble that of **10a**. However, since the C3 position in **11a** and 1**1b** accepted one H atom from the methyl group at the C2 position, respectively, and thus became *sp^3^*-hybridized, the torsion angles C4–C3–N2–B2 (**11a**: 94.25°; **11b**: 94.69°) are notably greater than those at the other three carbon positions (57–63°) in the central six-membered ring. Due to the disordered nature of the crystal, the bond lengths of **11a** and **11b** cannot be further discussed.

**Scheme 3:**
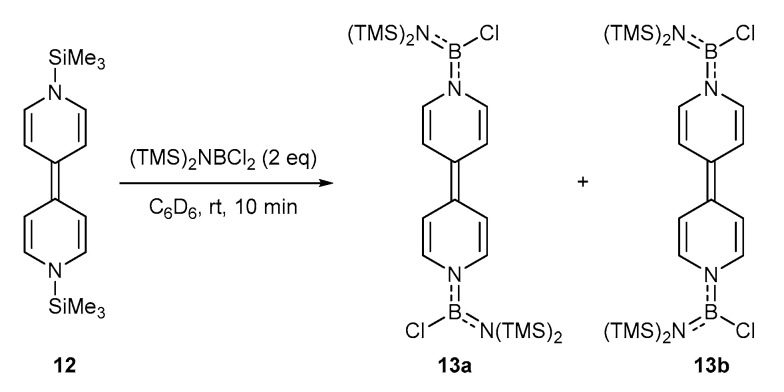
Synthesis of **13**.

**Figure 3:**
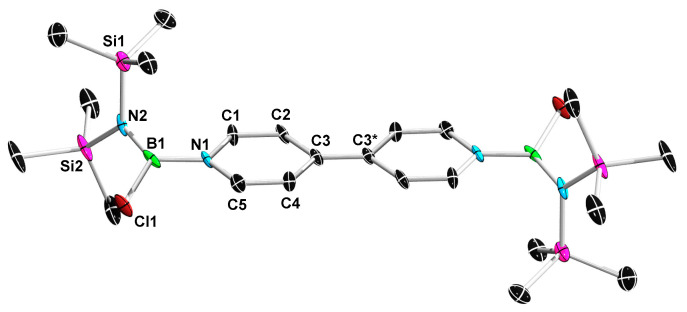
Molecular structure of **13a** in the solid state (ellipsoids set at 50% probability). Selected bond lengths (Å) and angles (°) for **13a**: B1–N1 1.459(8), B1–N2 1.392(10), Si1–N2 1.757(5), Si2–N2 1.770(5), N1–C1 1.402(8), N1–C5 1.414(8), C1–C2 1.332(8), C4–C5 1.347(9), C2–C3 1.452(9), C3–C4 1.444(9), C3–C3* 1.376(12), C1–N1–C5 115.7(5), C2–C1–N1 123.3(6), C1–C2–C3 122.9(6), C4–C3–C2 112.7(5), C5–C4–C3 123.3(6), C4–C5–N1 122.1(6).

The reaction of (SiMe_3_)_2_NBCl_2_ with **12** [38] of greater reducing power (redox potential of −0.40 V) [39] was performed at ambient temperature in C_6_D_6_. After the removal of the solvent and extraction with hexane, an NMR spectroscopically pure product **13** was obtained with a yield of 75%. Compound **13** was confirmed by NMR spectroscopic (Figures S29–S31) and HRMS studies. Suitable single crystals of **13a** for X-ray diffraction analysis were obtained upon storage of the reaction mixture overnight at RT (Figure 3 and Figure S41). The N1–C1/N1–C5 (1.402(8)–1.414(8) Å), C1–C2/C5–C4 (1.332(8)–1.347(9) Å), C2–C3/C3–C4 (1.444(9)–1.452(9) Å), C3–C3* (1.376(12) Å) distances are in line with the Lewis structure depicted in Scheme 3.

**Figure 4:**
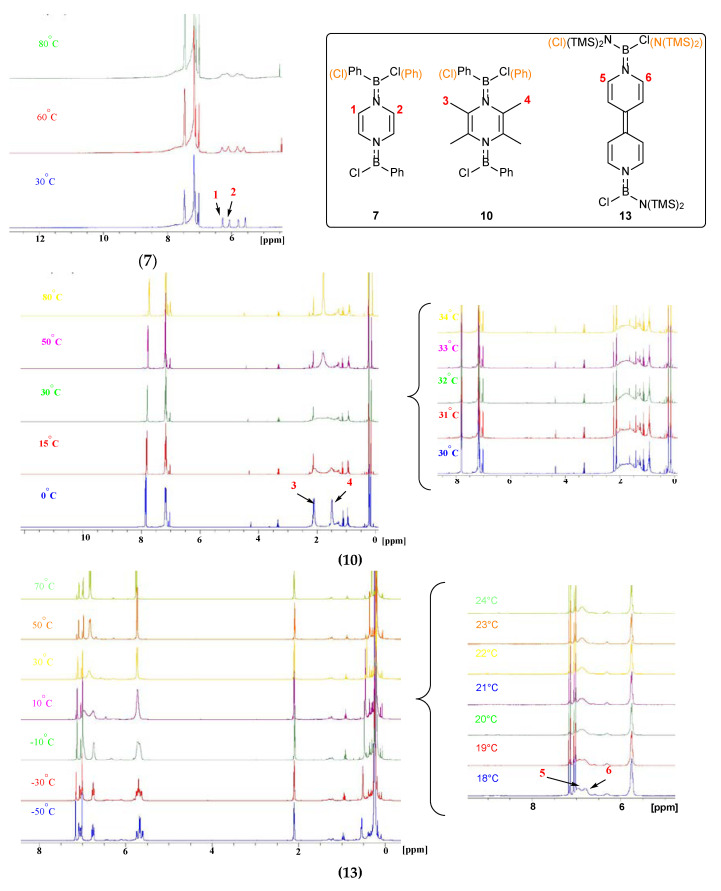
Variable-temperature ^1^H-NMR (400 MHz, toluene-d^8^) spectra of **7**, **10**, and **13**.

Apparently, both the RT-NMR spectroscopic and crystallographic studies failed to prove any successful reduction of the trivalent borane to divalent boron radical. Since the rotational barrier around an N―B dative bond should be lower than that of a B=N double bond, we assumed that any contribution from the bonding mode **B** (Scheme 1) should slightly lower the rotational barrier around the exocyclic B–N bond. In this context, we conducted a variable-temperature ^1^H-NMR experiment to provide further insight. Toluene-d^8^ was selected as the solvent with a temperature ranging from −60 °C to 80 °C. In general, the exocyclic H or Me as marked in Figure 4 (top right) should display two signals if the B=N bond is nonrotatable. The separated signals will coalesce at an elevated temperature when the B–N bond overcomes the rotational barrier and begins to rotate. Determination of the separation (Hz) of two signals and the coalescent temperature allows calculation of the B–N rotational barrier. The results of the VT-NMR experiments and assignment of the signals of interest are depicted in Figure 4 and Figures S32–S36. The obtained ΔG^≠^ values are summarized in Table 1. Analysis of the VT-NMR spectra revealed **7** with a strong B=N bond, and **10** and **13** with weak B=N bonds, as reflected by their rotational barriers >71.56 kJ/mol (**7**), 58.79 kJ/mol (**10**), 58.65 kJ/mol (**13**) when compared with ordinary B=N double bonds (71–100 kJ/mol) [40]. When taking the aforementioned assumption into account, the remarkably lower B–N rotational barrier of **10** compared to that of **7** is not in line with the fact that the reducing power of **2** is slightly weaker than that of **1**, according to the CV data. Therefore, the lower B–N rotational barrier in **10** should be mainly due to its boat conformation, which allows for less steric hindrance. Furthermore, although the central C_4_N_2_ rings of **7** and **13** both adopt a planar structure, the B–N rotational barrier in **13** is significantly lower than that of **7**. This finding could be explained by the competition in π donation from another *B*-amino function (–N(SiMe_3_)_2_) in **13**.

## 3. Materials and Methods

### 3.1. General Information

All manipulations were performed under dry argon using standard Schlenk line or glovebox techniques. Solvents were purified by distillation from Na under dry argon. C_6_D_6_ was dried over an Na/K alloy and then degassed by freeze–pump–thaw cycles. PhBCl_2_ was purchased from Beijing MREDA Technologie Co., Ltd., without any special treatment before use. The NMR spectra were acquired on a Bruker AVANCE 400 (^1^H: 400 MHz, ^13^C{^1^H}: 101 MHz, ^11^B: 128 MHz) NMR spectrometer at 298 K. Variable-temperature NMR experiments were conducted on a Bruker AVANCE 400 NMR spectrometer (^1^H: 400 MHz, 213–353 K). Chemical shifts are given in ppm. ^1^H and ^13^C{^1^H} NMR spectra were referenced to an external tetramethylsilane (TMS) via the residual protons of the solvent (^1^H) or the solvent itself (^13^C{^1^H}). ^11^B NMR spectra were referenced to the external BF_3_·OEt_2_. High-resolution mass spectrometry (HMRS) was performed with a Thermo Fisher Scientific Q Exactive Mass Spectrometer (MS) system.

### 3.2. Synthesis of **3** and **4**:

In the glove box, DurBBr_2_ (30.3 mg, 0.1 mmol, 1.0 equiv.) and 1,4-bis(trimethylsilyl)-1,4-diaza-2,5-cyclohexadiene (**1**) (22.6 mg, 0.1 mmol, 1.0 equiv.) were added into C_6_D_6_ (0.6 mL) in a J. Young NMR tube. The mixture was rested for 10 min prior to the removal of the volatiles under vacuum to get **3** as a pale yellow solid (23.6 mg, 72 mmol, 63%). Compound **4** was synthesized in a similar manner, with a yield of 64%.

**3**: **^1^H**-**NMR** (400 MHz, C_6_D_6_): δ = 6.86 (s, 1H, H of Dur), 6.35 (d, *J* = 6.6 Hz, 1H, H of C_4_N_2_), 5.13 (d, *J* = 6.5 Hz, 1H, H of C_4_N_2_), 4.91 (d, *J* = 6.6 Hz, 1H, H of C_4_N_2_), 4.64 (d, *J* = 6.5 Hz, 1H, H of C_4_N_2_), 2.29 (s, 6H, Me of Dur), 2.06 (s, 6H, Me of Dur), −0.23 (s, 9H, Me of TMS). **^13^C{^1^H}**-**NMR** (101 MHz, C_6_D_6_): δ = 134.5, 133.4, 132.0, 119.8, 118.7, 113.5, 112.8, 19.4, 18.4, −2.3 (9C, C of TMS). The carbon atom directly attached to boron was not detected, likely due to quadrupolar broadening. **^11^B**-**NMR** (128 MHz, C_6_D_6_): δ = 34.1. **HRMS**: calc. for [M]^+^ C_17_H_26_BBrN_2_Si^+^ 376.11362; found: 376.11308.

**4**: **^1^H**-**NMR** (400 MHz, C_6_D_6_): δ = 6.75 (s, 2H, H of Mes), 6.17 (d, *J* = 6.6 Hz, 1H, H of C_4_N_2_), 5.07 (d, *J* = 6.5 Hz, 1H, H of C_4_N_2_), 4.91 (d, *J* = 6.6 Hz, 1H, H of C_4_N_2_), 4.65 (d, *J* = 6.5 Hz, 1H, H of C_4_N_2_), 2.37 (s, 6H, Me of Mes), 2.15 (s, 3H, Me of Mes), 0.21 (s, 9H, Me of TMS). **^13^C{^1^H}**-**NMR** (101 MHz, C_6_D_6_): δ = 139.2, 137.8, 127.5, 119.4, 118.5, 112.4 (1C, C of C_4_N_2_), 112.8 (1C, C of C_4_N_2_), 21.3 (2C, *o*-CH_3_ of Mes), 20.9 (C, *p*-CH_3_ of Mes), −2.3 (9C, C of TMS). The carbon atom directly attached to boron was not detected, likely due to quadrupolar broadening. **^11^B**-**NMR** (128 MHz, C_6_D_6_): δ = 34.2. **HRMS**: calc. for [M]^+^ C_16_H_24_BClN_2_Si^+^ 318.14848; found: 318.14877.

### 3.3. Synthesis of **5**–**7**

In the glove box, DurBBr_2_ (60.4 mg, 0.2 mmol, 2.0 equiv.) and 1,4-bis(trimethylsilyl)-1,4-diaza-2,5-cyclohexadiene (**1**) (22.6 mg, 0.1 mmol, 1.0 equiv.) were added into C_6_D_6_ (0.6 mL) in a J. Young NMR tube. The mixture was rested overnight prior to the removal of the volatiles under vacuum to get **5** as a yellow oil with a 45% yield. Mixture **5** contains the *cis*-structure **5a** and *trans*-structure **5b**, and the ratio of the *cis-trans* isomers was about 1:1. Compounds **6**–**7** were synthesized in a similar manner, with a *cis-trans* isomers ratio of about 1:1 (yield: 52% (**6**) and 48% (**7**)].

**5a** + **5b**: **^1^H**-**NMR** (400 MHz, C_6_D_6_): δ = 6.86 (s, 2H), 6.82 (s, 2H), 6.52 (s, 2H), 6.26 (d, ^3^*J*_H-H_ = 1.6 Hz, 1H), 6.24 (d, ^3^*J*_H-H_ =1.6 Hz, 1H), 5.36 (d, ^3^*J*_H-H_ = 1.6 Hz, 1H), 5.34 (d, ^3^*J*_H-H_ = 1.6 Hz, 1H), 4.99 (s, 2H), 2.09 (s, 12 H), 2.07 (s, 12 H), 2.03 (s, 12 H), 2.01 (s, 12H). **^13^C{^1^H}**-**NMR** (101 MHz, C_6_D_6_): δ = 134.0, 133.9, 133.6, 133.5, 132.5, 132.3, 118.2, 117.7, 117.2, 116.6, 19.2, 19.1, 18.5, 18.4. The carbon atom directly attached to boron was not detected, likely due to quadrupolar broadening. **^11^B**-**NMR** (128 MHz, C_6_D_6_): δ = 38.9. **HRMS**: calc. for [M]^+^ C_24_H_30_N_2_B_2_Br_2_^+^ 526.09564; found: 526.09549.

**6a** + **6b**: **^1^H**-**NMR** (400 MHz, C_6_D_6_): δ = 6.69 (s, 4H), 6.67 (s, 4H), 6.34 (s, 2H), 6.09 (d, ^3^*J*_H-H_ = 1.6 Hz, 1H), 6.08 (d, ^3^*J*_H-H_ = 1.6 Hz, 1H), 5.32 (d, ^3^*J*_H-H_ = 1.6 Hz, 1H), 5.30 (d, ^3^*J*_H-H_ = 1.6 Hz, 1H), 4.94 (s, 2H), 2.19 (s, 12H), 2.16 (s, 12H), 2.13 (s, 6H), 2.12 (s, 6H).**^13^C{^1^H}**-**NMR** (101 MHz, C_6_D_6_): δ = 138.9, 138.8, 138.6, 138.5, 127.6, 127.6, 117.3, 116.4, 116.3, 115.5, 21.2, 21.1, 20.9, 20.8. The carbon atom directly attached to boron was not detected, likely due to quadrupolar broadening. **^11^B**-**NMR** (128 MHz, C_6_D_6_): δ = 38.5. **HRMS**: calc. for [M]^+^ C_22_H_26_N_2_B_2_Cl^+^ 410.16537; found: 410.16492.

**7a** + **7b**: **^1^H**-**NMR** (400 MHz, C_6_D_6_): δ = 7.86 (s, 2 H), 7.51−7.46 (m, 8 H), 7.19−7.15 (m, 10 H), 6.29 (s, 2H), 6.08 (d, ^3^*J*_H-H_ = 1.68 Hz, 1H), 6.06 (d, ^3^*J*_H-H_ = 1.64 Hz, 1H), 5.78 (d, ^3^*J*_H-H_ = 1.60 Hz, 1H), 5.76 (d, ^3^*J*_H-H_ = 1.70 Hz, 1H), 5.54 (s, 2 H). **^13^C{^1^H}**-**NMR** (101 MHz, C_6_D_6_): δ = 133.3, 133.2, 130.2, 130.1, 127.9, 127.8, 118.0, 117.5, 116.8, 116.5. The carbon atom directly attached to boron was not detected, likely due to quadrupolar broadening. **^11^B**-**NMR** (128 MHz, C_6_D_6_): δ = 36.5. **HRMS**: calc. for [M]^+^ C_16_H_14_N_2_B_2_Cl_2_^+^ 326.07147; found: 326.07069.

### 3.4. Synthesis of **8** and **9**

Compounds **8** and **9** were synthesized in a similar manner as **3** and **4**, with yields of 65% and 75%, respectively.

**8**: **^1^H-NMR** (400 MHz, C_6_D_6_): δ = 7.93–7.90 (m, 2H), 7.85–7.83 (m, 1H), 7.24–7.13 (m, 2H), 2.14 (s, 3H), 1.68 (s, 3H), 1.62 (s, 3H), 1.52 (s, 3H), 0.19 (s, 9H). **^13^C{^1^H}**-**NMR** (101 MHz, C_6_D_6_): δ = 132.5, 132.2, 131.9, 131.9, 131.6, 128.6, 128.0, 122.8, 121.8, 16.8, 16.7, 16.7, 16.5, 0.2. The carbon atom directly attached to boron was not detected, likely due to quadrupolar broadening. **^11^B**-**NMR** (128 MHz, C_6_D_6_): δ = 35.9. **HRMS**: calc. for [M+H]^+^ C_17_H_27_N_2_BClSi^+^ 333.17196; found: 333.17233.

**9**: **^1^H**-**NMR** (400MHz, C_6_D_6_): δ = 6.88 (s, 1H), 2.43 (s, 3H), 2.29 (s, 3H), 2.24 (s, 3H, Me of Dur), 2.10 (s, 3H), 2.06 (s, 3H), 1.71 (s, 3H), 1.48 (s, 3H), 1.44 (s, 3H), 0.25 (s, 9H). **^13^C{^1^H}**-**NMR** (101 MHz, C_6_D_6_): δ = 133.8, 133.2, 133.1, 133.0, 132.8, 131.6, 131.4, 122.5, 122.0, 19.6, 19.4, 19.3, 19.3, 19.2, 18.1, 18.0, 16.1, 1.8. The carbon atom directly attached to boron was not detected, likely due to quadrupolar broadening. **^11^B**-**NMR** (128 MHz, C_6_D_6_): δ = 37.7. **HRMS**: calc. for [M+H]^+^ C_21_H_35_N_2_BBrSi^+^ 433.18405; found: 433.18483.

### 3.5. Synthesis of **10**

In the glove box, PhBCl_2_ (31.6 mg, 0.2 mmol, 2.0 equiv.) and 2,3,5,6-tetramethyl-1,4-bis(trimethylsilyl)-1,4-diaza-2,5-cyclohexadiene (**2**) (28.2 mg, 0.1 mmol, 1.0 equiv.) were added into C_6_D_6_ (0.6 mL) in a J. Young NMR tube. The mixture was rested overnight prior to the removal of the volatiles under vacuum to get **10** as a pale yellow solid (24.9 mg, 0.53 mmol, 53%).

**10a** + **10b**: **^1^H**-**NMR** (400 MHz, toluene-d^8^): δ = 7.72–7.20 (m, 8H), 7.11–7.06 (m, 12H), 1.95 (br, 12H), 1.45 (br, 12H). **^13^C{^1^H}**-**NMR** (101 MHz, toluene-d^8^): δ = 133.2, 130.2, 129.1, 128.6, 128.2, 128.0, 127.9, 127.7, 125.4, 124.9, 20.8, 20.7, 20.3, 20.1. The carbon atom directly attached to boron was not detected, likely due to quadrupolar broadening. **^11^B**-**NMR** (128 MHz, toluene-d^8^): δ = 36.9. **HRMS**: calc. for [M+H]^+^ C_20_H_23_N_2_B_2_Cl_2_^+^ 383.14189; found: 383.14083.

### 3.6. Synthesis of **11a** and **11b**

In the glove box, **9** (37.6 mg, 0.1 mmol, 1 equiv.) and DurBBr_2_ (30.3 mg, 1 mmol, 1.0 equiv.) were added into C_6_D_6_ (0.6 mL) in a J. Young NMR tube. The mixture was rested overnight prior to the removal of the volatiles under vacuum to get **11** as a yellow oil with a yield of 76%. Mixture **11** contains two olefin isomers, **11a** and **11b**, with a ratio of about 1:0.3.

**11a**: **^1^H**-**NMR** (400 MHz, C_6_D_6_): δ = 6.91 (s, 1H), 6.89 (s, 1H), 5.14–5.13 (m, 1H), 4.72–4.71 (m, 1H), 4.38 (s, 1H), 2.58 (s, 3H), 2.55 (s, 3H), 2.35 (s, 3H), 2.32 (s, 3H), 2.11 (s, 3H), 2.09 (s, 6H), 2.07 (s, 3H), 1.99 (d, ^3^*J*_H-H_ = 0.8 Hz, 3H), 1.41 (d, ^3^*J*_H-H_ = 0.8 Hz, 3H), 0.91 (d, ^3^*J*_H-H_ = 6.6 Hz, 3H).

**11b**: **^1^H**-**NMR** (400 MHz, C_6_D_6_): δ = 6.89 (s, 1H), 6.88 (s, 1H), 5.75–5.70 (m, 1H), 4.47–4.46 (m, 1H), 4.07–4.06 (m, 1H), 2.54 (s, 3H), 2.52 (s, 3H), 2.36 (s, 3H), 2.33 (s, 3H), 2.10 (s, 3H), 2.09 (s, 9H), 2.07 (s, 3H), 1.98 (d, ^3^*J*_H-H_ = 1.1 Hz, 3H), 1.45 (d, ^3^*J*_H-H_ = 1.1 Hz, 3H), 1.13 (d, ^3^*J*_H-H_ = 8.0 Hz, 3H).

**11a** + **11b**: **^13^C{^1^H}**-**NMR** (101 MHz, C_6_D_6_): δ = 152.0, 151.4, 133.7, 133.6, 133.6, 133.5, 133.4, 133.3, 133.2, 133.2, 132.6, 132.2, 132.1, 132.0, 131.7, 131.4, 131.4, 131.1, 130.8, 129.9, 105.6, 101.7, 61.2, 60.8, 22.8, 22.6, 20.2, 20.1, 20.0, 19.7, 19.7, 19.6, 19.5, 19.4, 19.3, 19.3, 19.2, 19.2, 19.2, 19.1, 18.9, 18.1, 17.9, 15.4. The carbon atom directly attached to boron was not detected, likely due to quadrupolar broadening. **^11^B-NMR** (128 MHz, C_6_D_6_): δ = 39.1. **HRMS**: calc. for [M+H]^+^ C_28_H_39_N_2_B_2_Br_2_^+^ 585.16402; found: 585.16454.

### 3.7. Synthesis of **13a** and **13b**

In the glove box, (TMS)_2_NBCl_2_ (86.4 mg, 0.2 mmol, 2 equiv.) and 1,1′-bis(trimethylsilyl)-1H,1′H-4,4′-bipyridinylidene (**12**) (30.2 mg, 0.1 mmol, 1 equiv.) were added into C_6_D_6_ (0.6 mL). The mixture was rested for 10 min prior to the removal of the volatiles under vacuum to get a yellowish green powder. The yellowish green powder was extracted with hexane, filtered, and the solvent was again removed under reduced pressure to yield **13** as a yellow powder (60.0 mg, 0.84 mmol, 84%). The ratio of **13a**:**13b** is ca. 1:1.

**13a** + **13b**: **^1^H**-**NMR** (400 MHz, C_6_D_6_): δ = 6.87 (br, 8H), 5.79 (d,^3^*J*_H-H_ = 8.1 Hz, 8H), 0.21 (s, 72H). **^13^C{^1^H}-NMR** (101 MHz, C_6_D_6_): δ = 114.3, 111.3, 2.25 (C of TMS). The carbon atom directly attached to boron was not detected, likely due to quadrupolar broadening. **^11^B**-**NMR** (128 MHz, C_6_D_6_): δ = 34.4. **HRMS**: calc. for [M]^+^ C_22_H_44_N_4_B_2_Cl_2_Si_4_^+^ 568.22007; found: 568.21887.

## 4. Conclusions

In summary, the reactions of electron-rich organosilicon compounds **1**, **2**, and **12** with various *B*-amino and *B*-aryl dihaloboranes were comprehensively studied. No direct evidence for the presence of divalent boron radical character could be obtained from NMR spectra and single-crystal structures. The rotational barrier around the exocyclic B–N bonds was studied by VT ^1^H-NMR spectroscopy, which revealed relatively small barriers for **10** and **13**. The steric hindrance as well as the competition from additional *B*-amino functions were the main factors affecting the B–N rotational barrier. In addition, the reaction between **2** and DurBBr_2_ resulted in **11** via an isomerization process. Although this study does not access the desired biradial species, we believe that the novel B=N-containing products could act as an RXB• source upon the liberation of the aromatic linker (i.e., pyrazine and 4,4′-bipyridine). Studies of the mechanism of the isomerization reaction, as well as the application of **10** and **13** as RXB• transfer reagents to unsaturated organic substrates, are currently underway in our laboratory, and will be reported in due course.

## Figures and Tables

**Table 1:** Rotational barrier of **7**, **10**, and **13**.

Compound	Tc	△ν	△G^≠^
**7**	>80 °C (353 K)	85.0 Hz	>71.56 kJ/mol
**10**	32 °C (305 K)	243.5 Hz	58.79 kJ/mol
**13**	20 °C (293 K)	96.0 Hz	58.65 kJ/mol

Tc = coalescence temperature; Δν = the separation in hertz between the two singlets in the absence of exchange; **∆G^≠^** = rotational barrier.

## Supplementary Materials

Supplementary materials are available online. Figures S1–S31: NMR spectra for **3**–**11**, **13**. Figures S32–S36: Variable-temperature ^1^H-NMR spectra for **7**, **10**, and **13**. Figures S37–S41, Single crystal structure for **5**, **9**–**11**, and **13**. Table S1: Crystal data for **5**, **9**–**11**, and **13**.
