# Supplementary material for: Reactions of Dihaloboranes with Electron-Rich 1,4-Bis(trimethylsilyl)-1,4-diaza-2,5-cyclohexadienes
Source: Molecules. 2020 Jun 22;25(12):2875. doi: 10.3390/molecules25122875 (PMC7356994; doi:10.3390/molecules25122875)
Supplement: Supplementary file 1 [file molecules-25-02875-s001.pdf]

Reactions of Dihaloboranes with Electron-Rich 1,4-Bis(trimethylsilyl)-1,4-diaza-2,5-cyclohexadienes

Li Ma[†], Xiaolin Zhang[†], Wenbo Ming[†], Shengxin Su, Xiaoyong Chang, Qing Ye*

Department of Chemistry, Southern University of Science and Technology, 518055 Shenzhen, P. R. China

* Correspondence: yeq3@sustech.edu.cn; Tel.: +86 (0)755-88018354

[†] These authors contributed equally to this work

Table of Contents

I. NMR Spectra of Products 2

II. Crystallographic details 20

I. NMR Spectra of Products

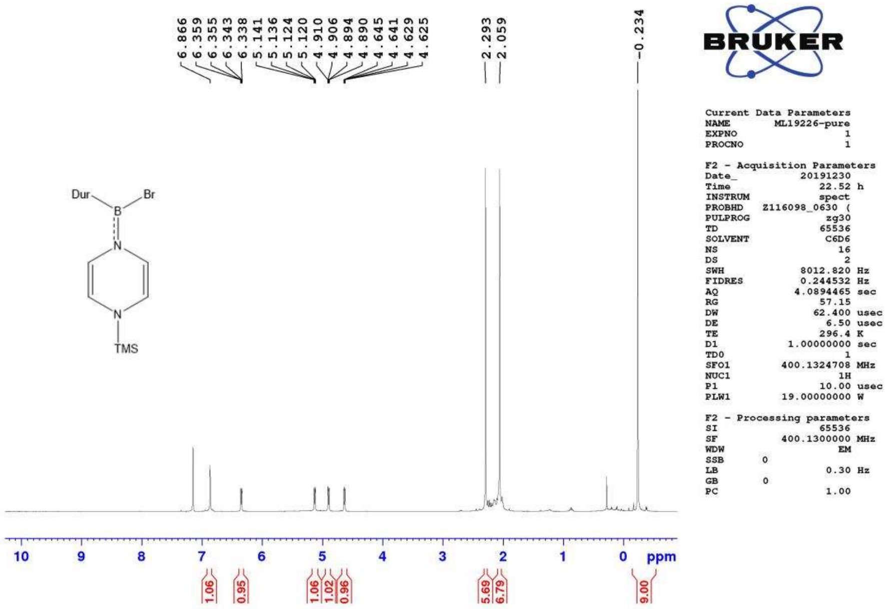

Figure S1. ^{1}H NMR (400 MHz, C_6D_6) of **3**

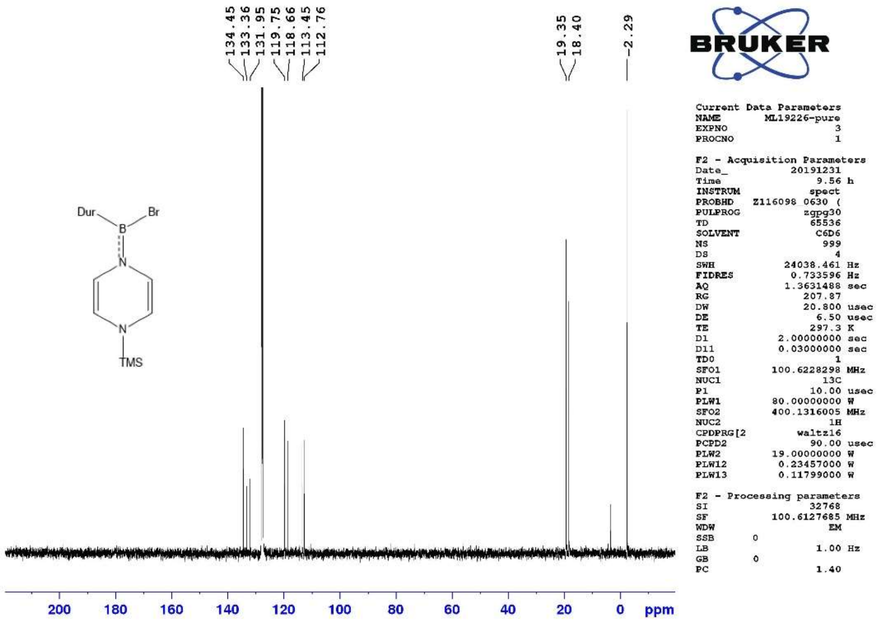

Figure S2. $^{13}C\{^{1}H\}$ NMR (101 MHz, C_6D_6) of **3**

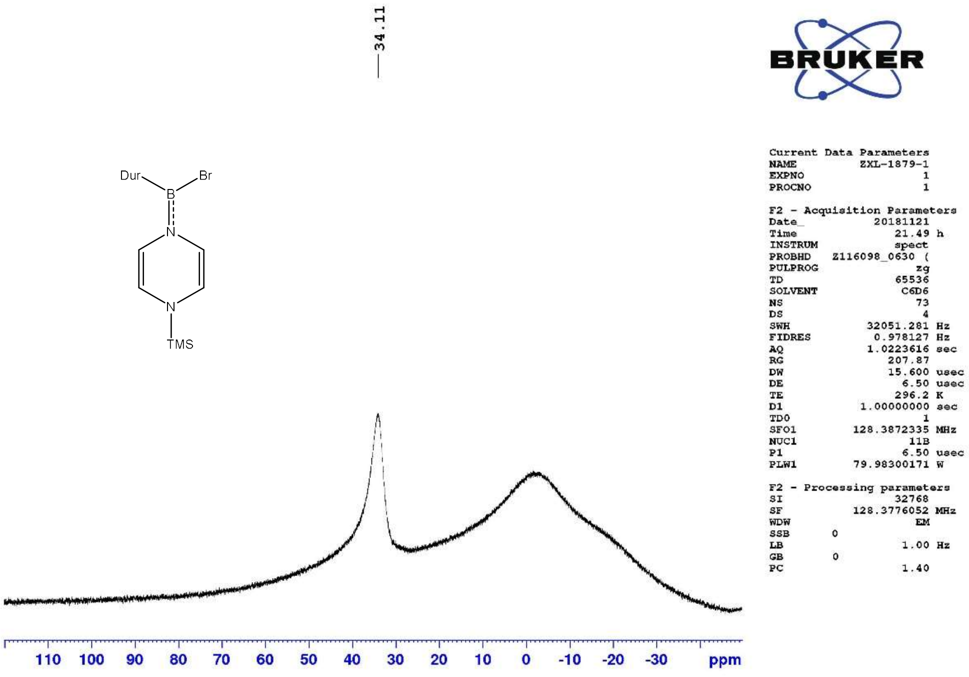

Figure S3. ^{11}B NMR (128 MHz, C_6D_6) of **3**

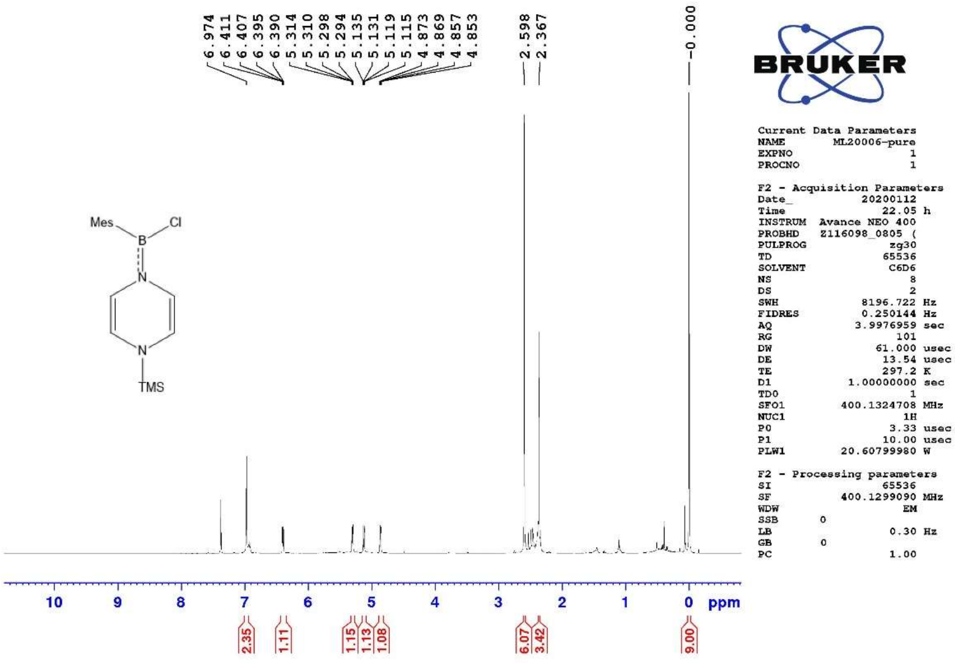

Figure S4. ^{1}H NMR (400 MHz, C_6D_6) of **4**

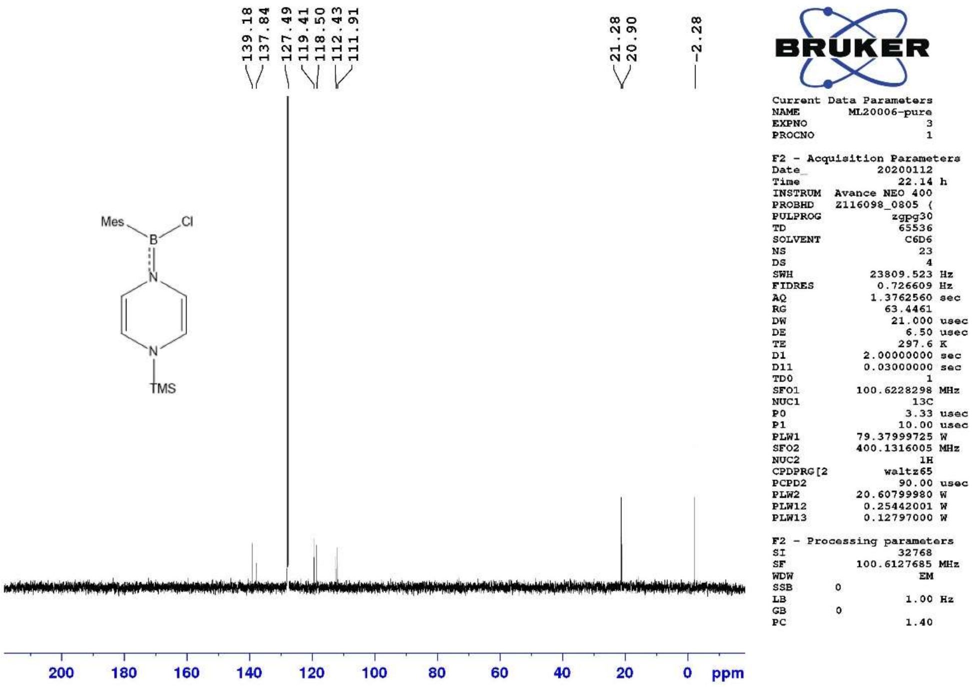

Figure S5. $^{13}C\{^{1}H\}$ NMR (101 MHz, C_6D_6) of **4**

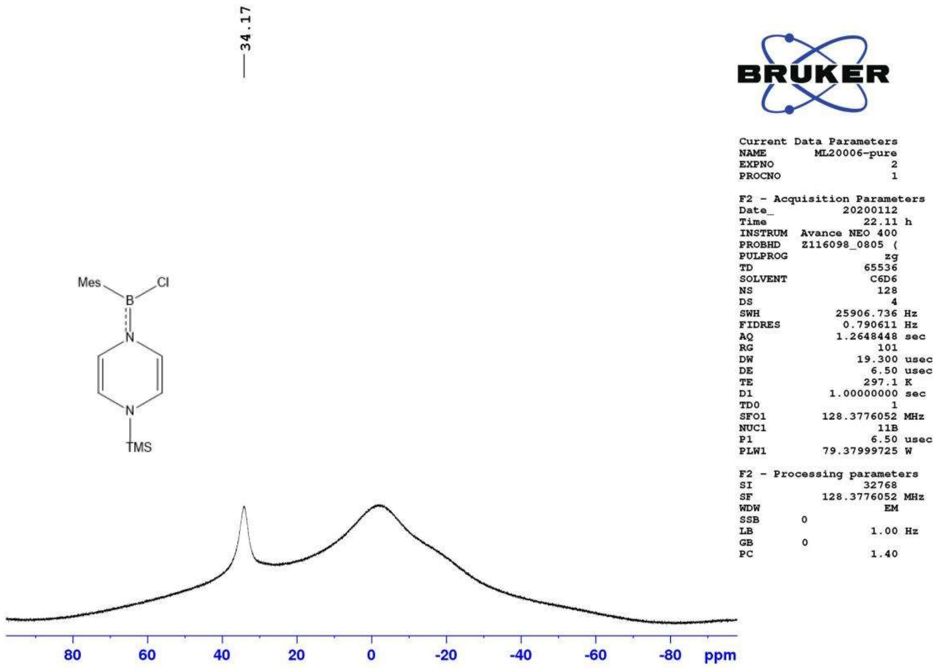

Figure S6. ^{11}B NMR (128 MHz, C_6D_6) of **4**

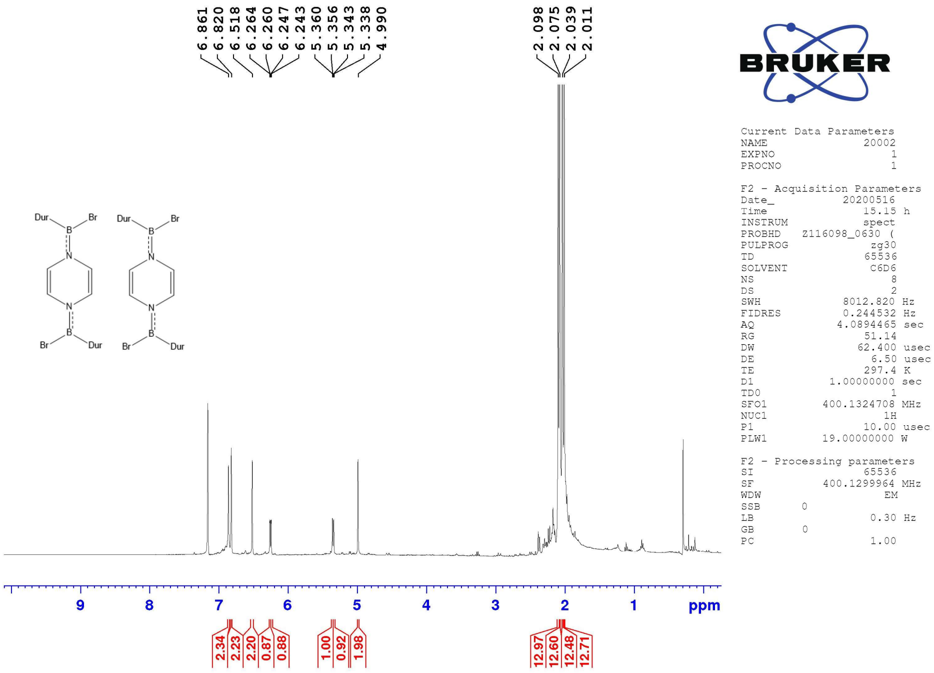

Figure S7. ^{1}H NMR (400 MHz, C_6D_6) of **5a** + **5b**

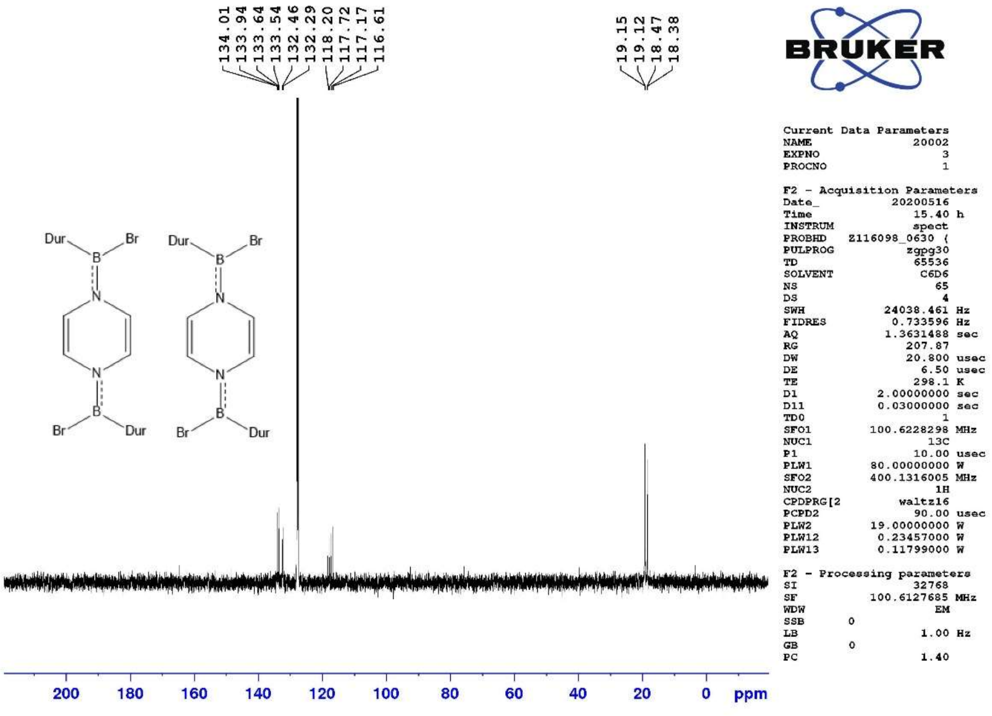

Figure S8. ^{13}C{^{1}H} NMR (101 MHz, C_6D_6) of **5a** + **5b**

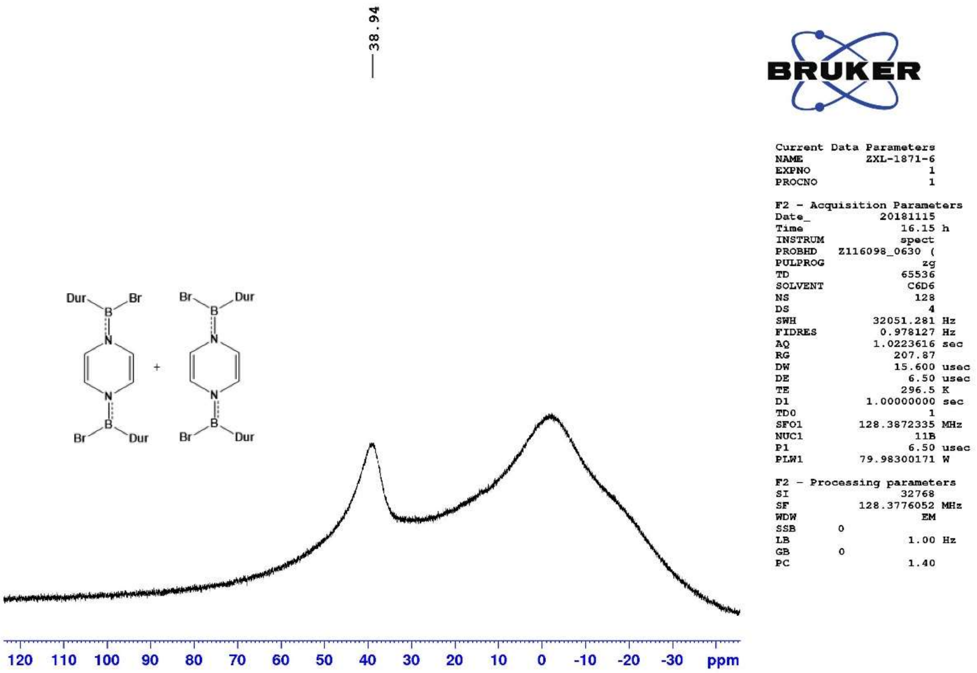

Figure S9. ^{11}B NMR (128 MHz, C_6D_6) of **5a** + **5b**

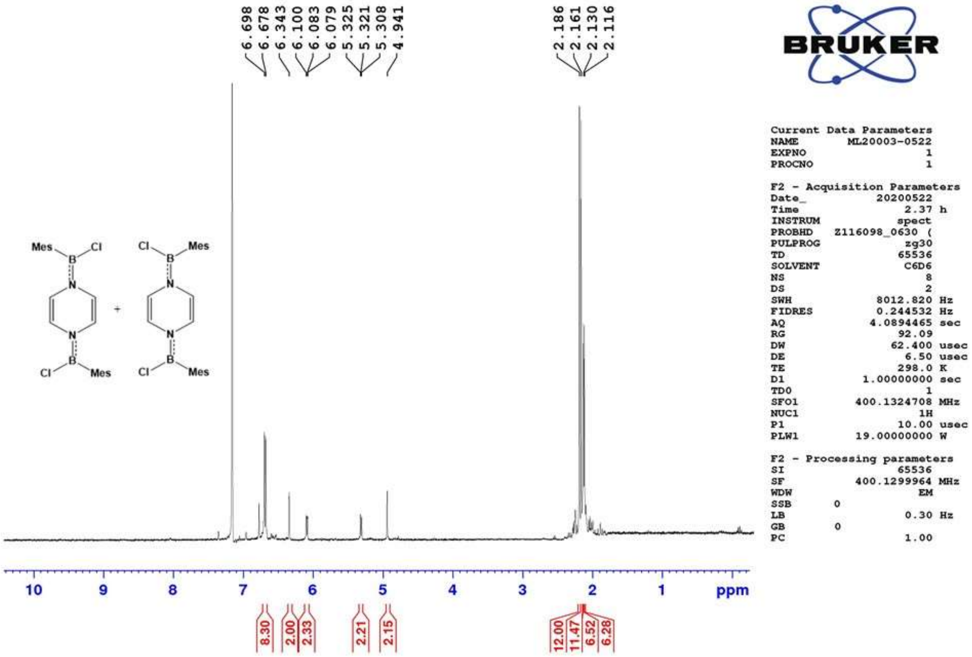

Figure S10. ^{1}H NMR (400 MHz, C_6D_6) of **6a** + **6b**

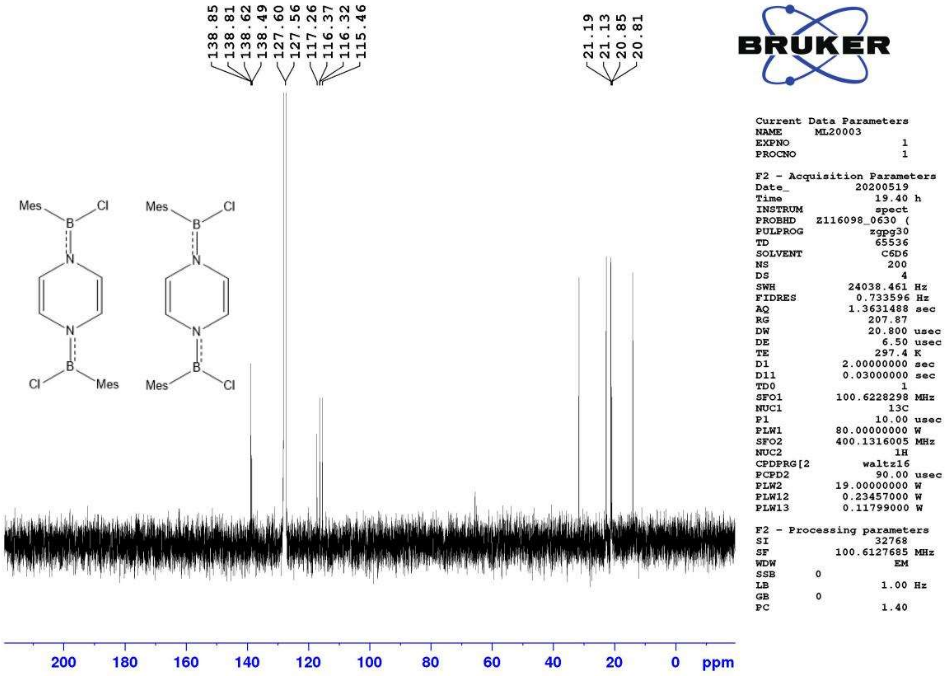

Figure S11. $^{13}C\{^{1}H\}$ NMR (101 MHz, C_6D_6) of **6a** + **6b**

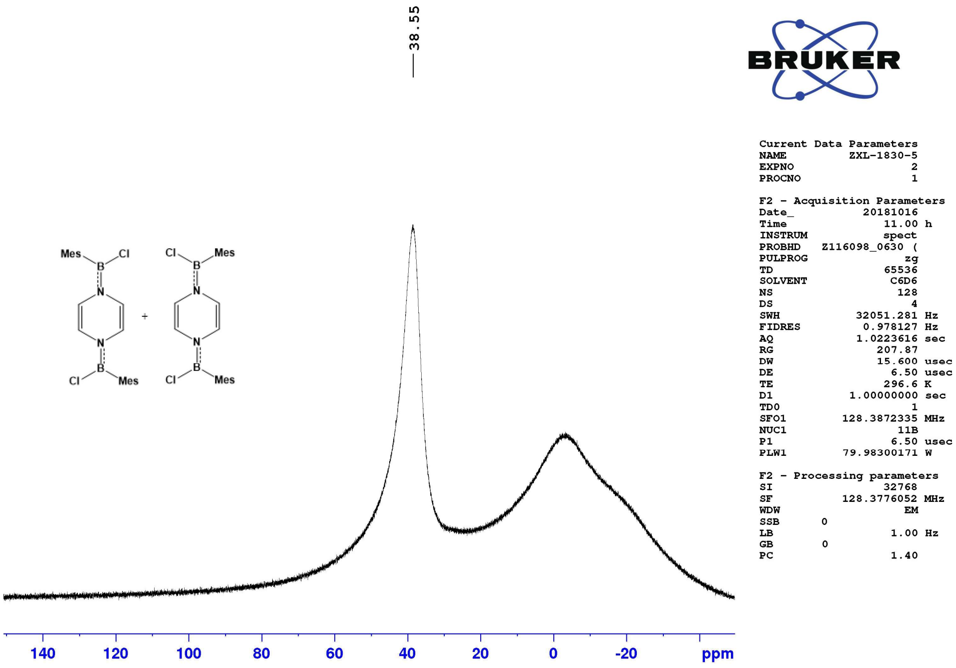

Figure S12. ^{11}B NMR (128 MHz, C_6D_6) of **6a** + **6b**

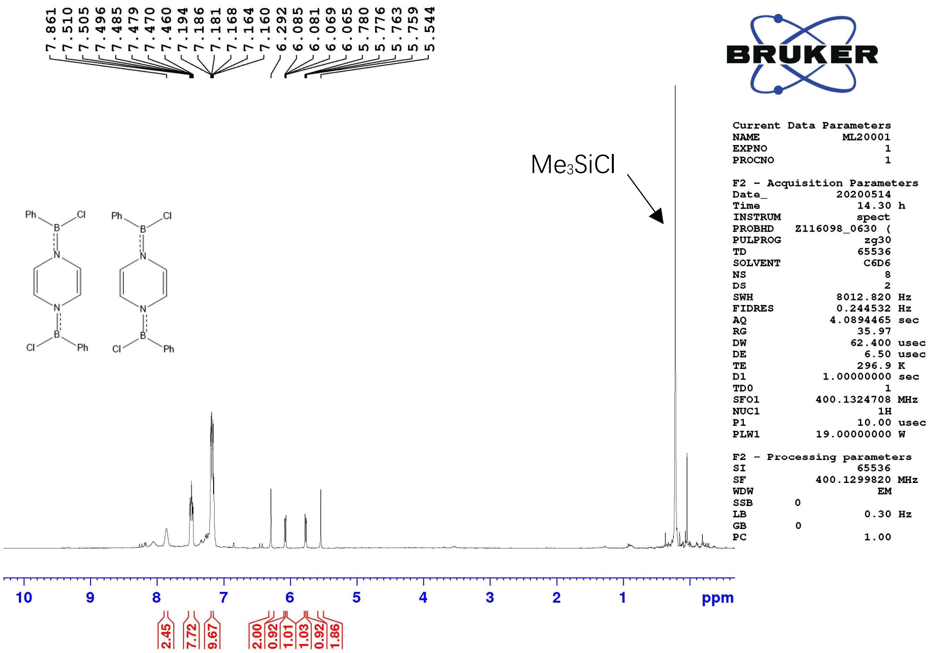

Figure S13. ^{1}H NMR (400 MHz, C_6D_6) of **7a** + **7b** (Complete removal of Me_3SiCl led to partial decomposition of **7a** and **7b**)

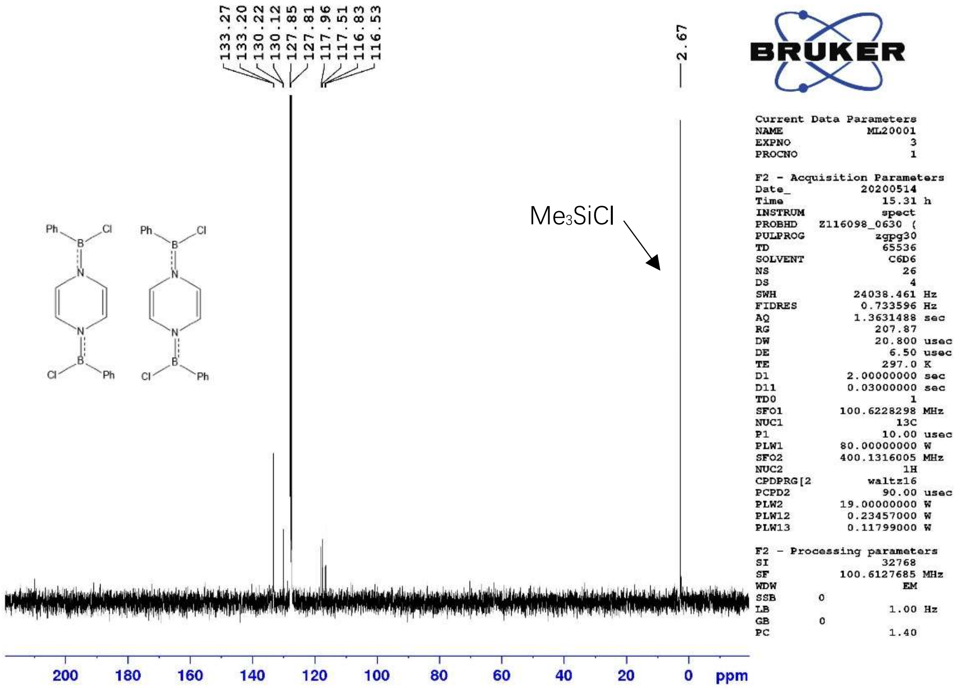

Figure S14. $^{13}C\{^{1}H\}$ NMR (101 MHz, C_6D_6) of **7a** + **7b** (Complete removal of Me_3SiCl led to partial decomposition of **7a** and **7b**)

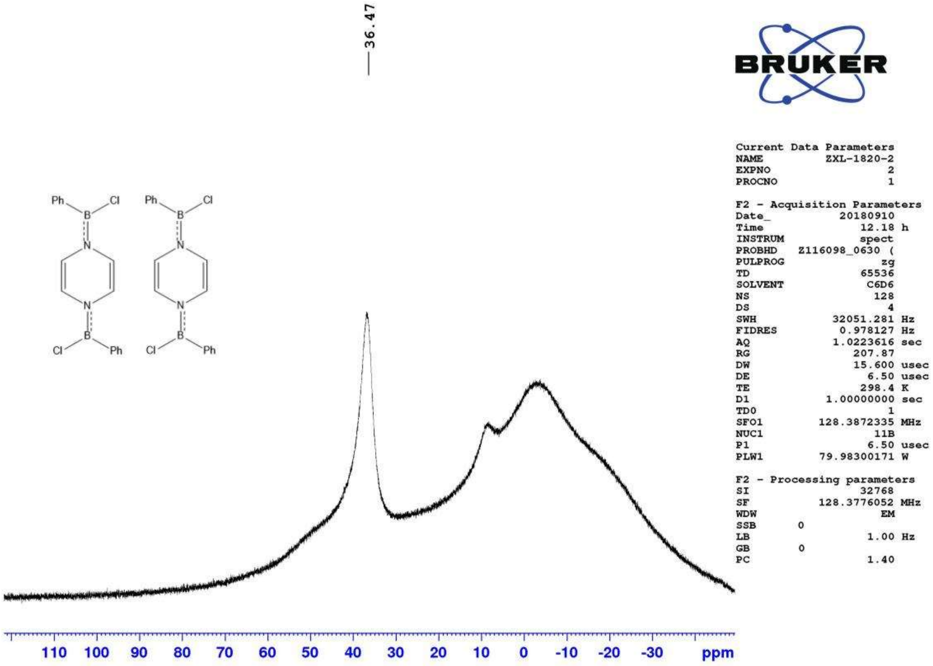

Figure S15. ^{11}B NMR (128 MHz, C_6D_6) of **7a** + **7b**

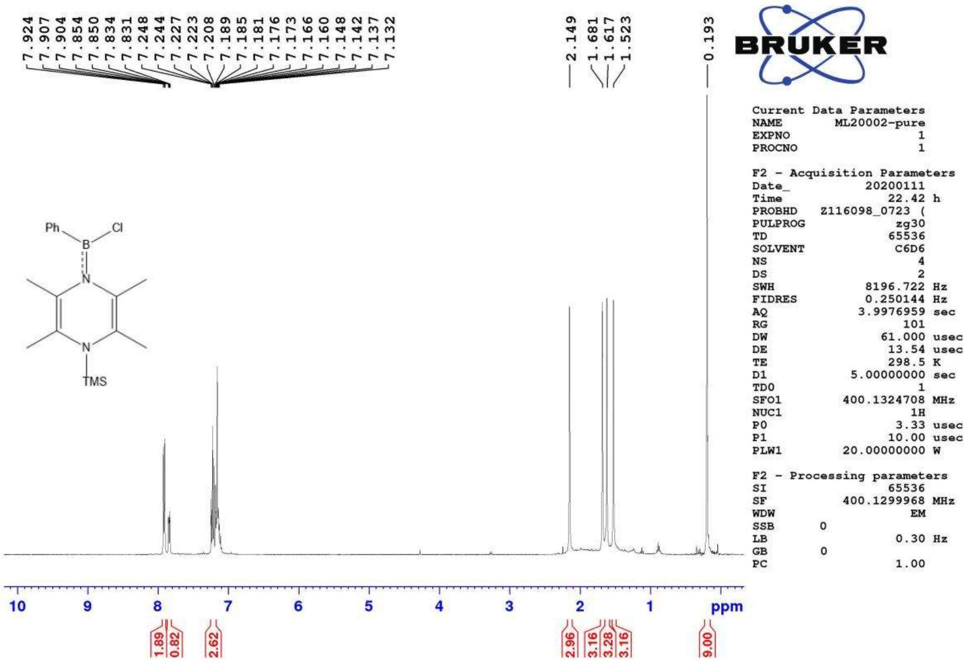

Figure S16. ^{1}H NMR (400 MHz, C_6D_6) of **8**

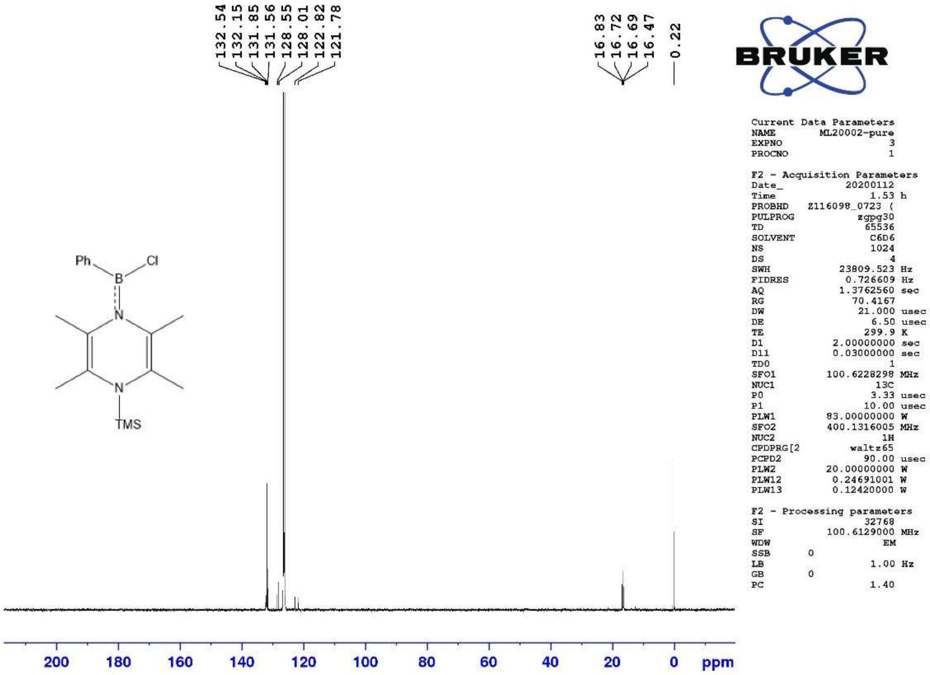

Figure S17. $^{13}C\{^{1}H\}$ NMR (101 MHz, C_6D_6) of **8**

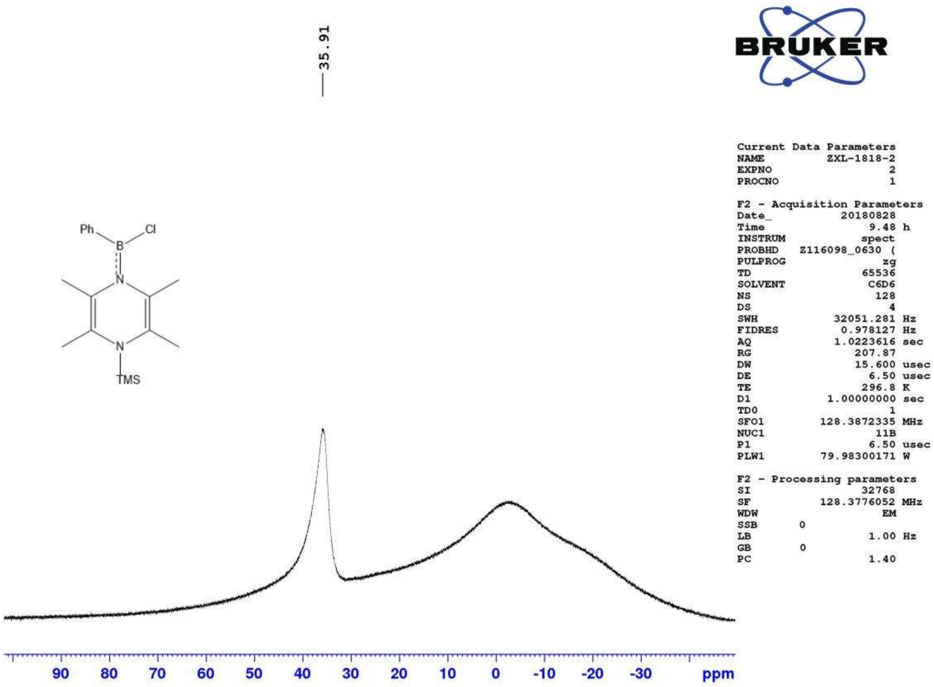

Figure S18. ^{11}B NMR (128 MHz, C_6D_6) of **8**

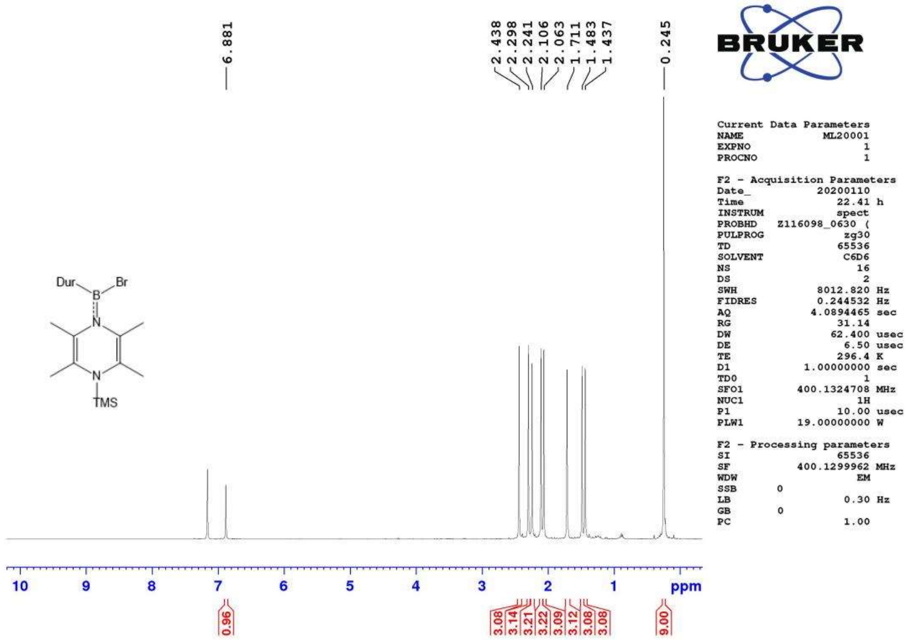

Figure S19. ^{1}H NMR (400 MHz, C_6D_6) of **9**

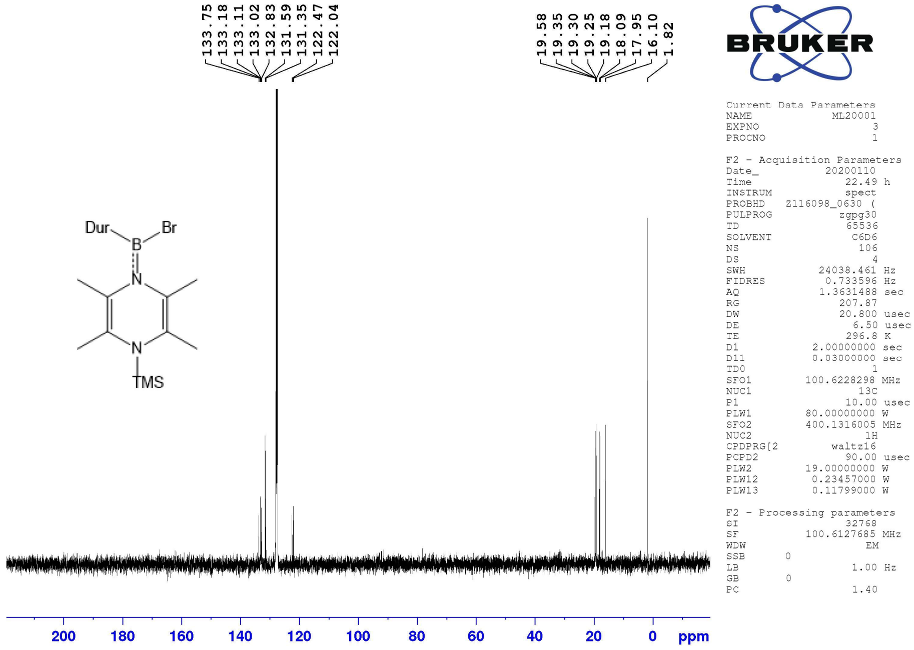

Figure S20. $^{13}C\{^{1}H\}$ NMR (101 MHz, C_6D_6) of **9**

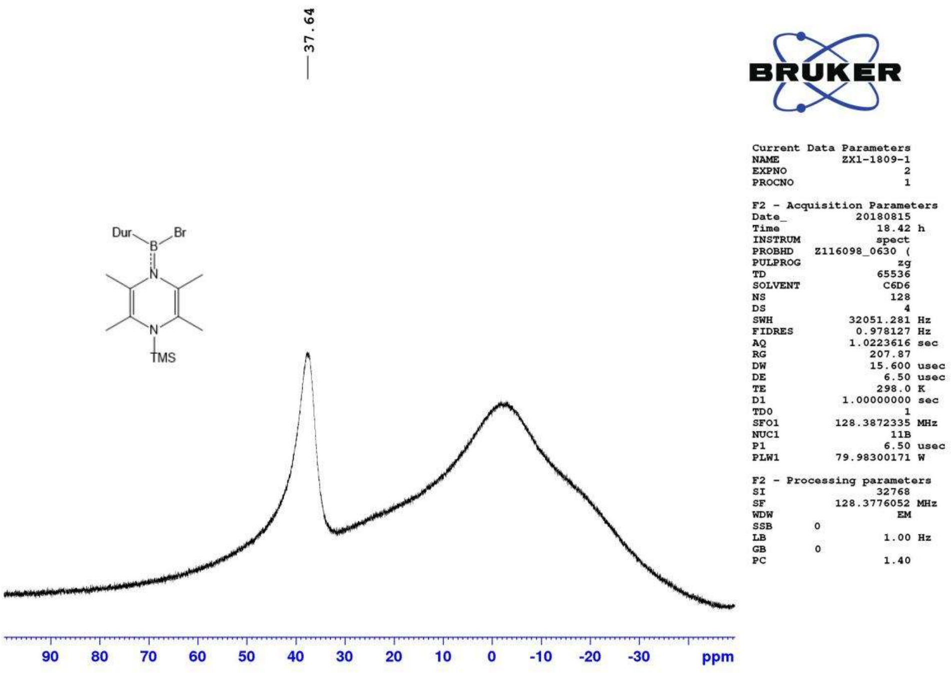

Figure S21. ^{11}B NMR (128 MHz, C_6D_6) of **9**

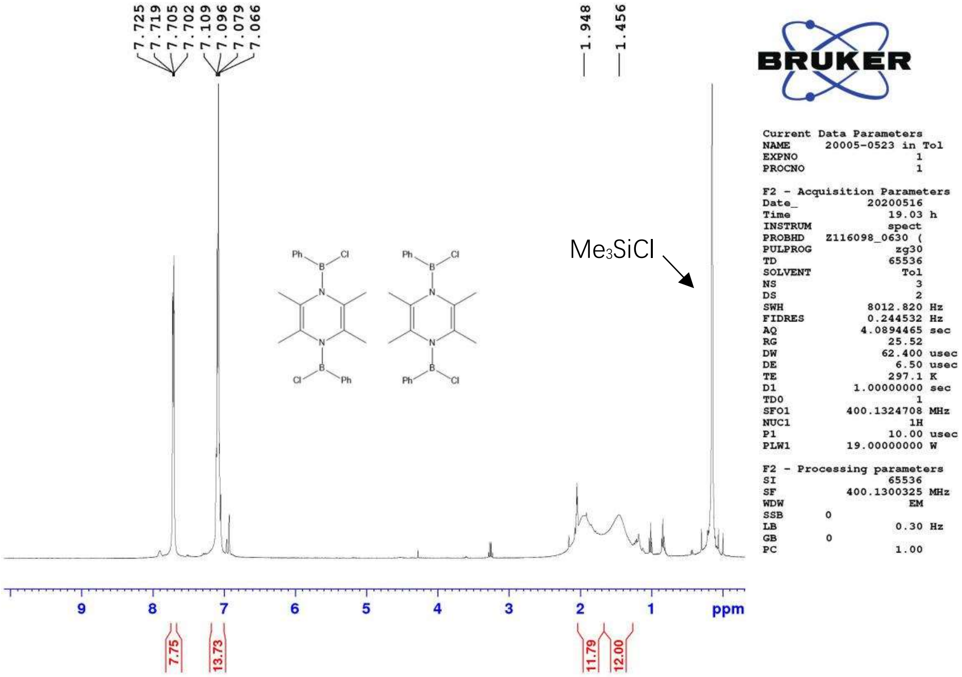

Figure S22. ^{1}H NMR (400 MHz, toluene-d^8) of **10a** + **10b** (Complete removal of Me_3SiCl led to partial decomposition of **10a** and **10b**)

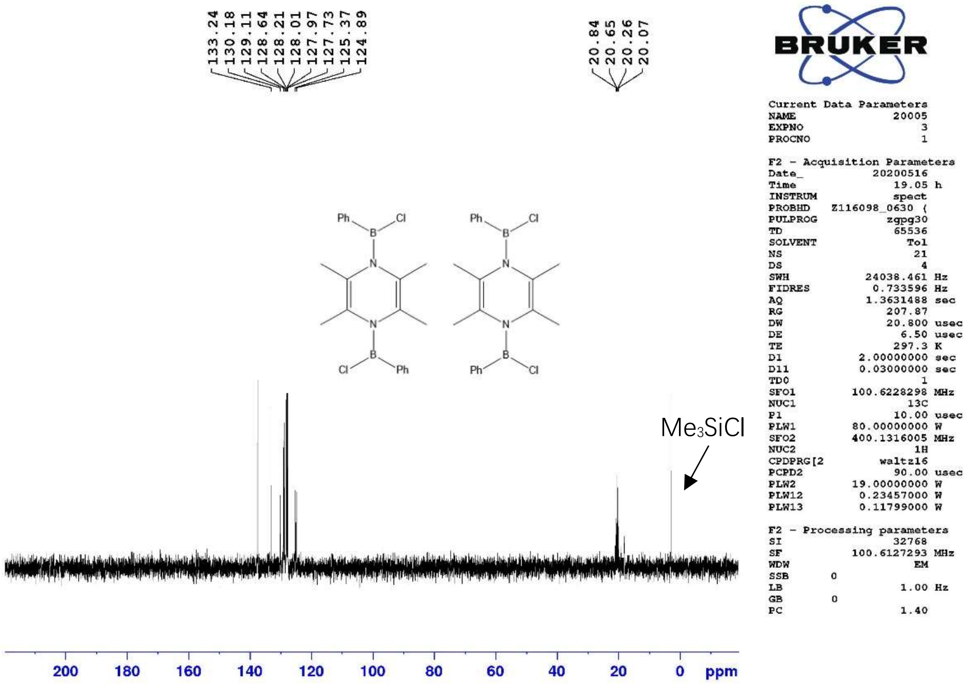

Figure S23. $^{13}C\{^{1}H\}$ NMR (101 MHz, toluene-d^8) of **10a** + **10b** (Complete removal of Me_3SiCl led to partial decomposition of **10a** and **10b**)

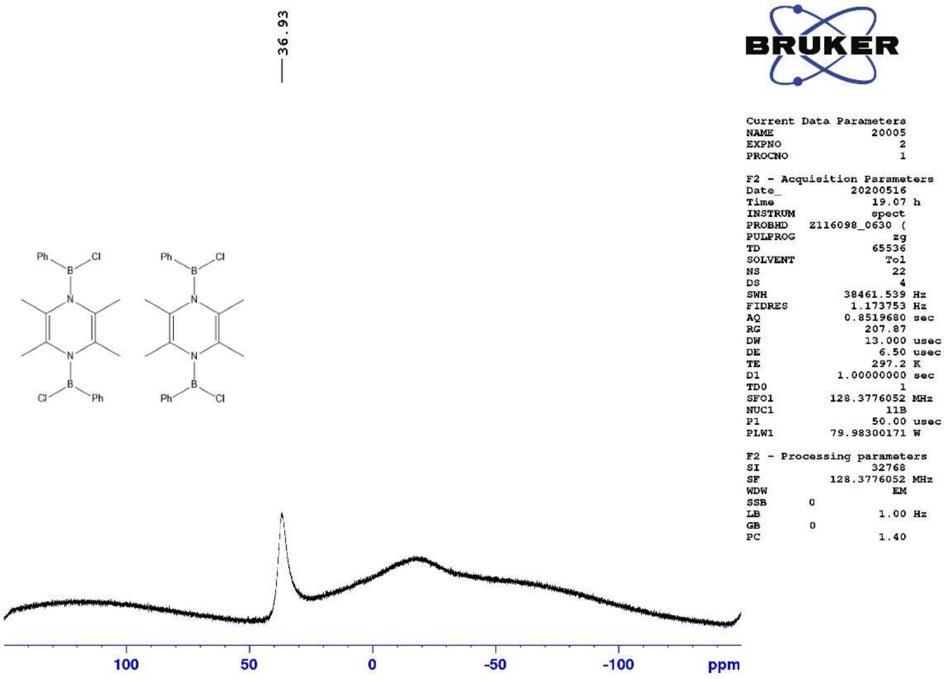

Figure S24. ^{11}B NMR (128 MHz, toluene-d^8) of **10a** + **10b**

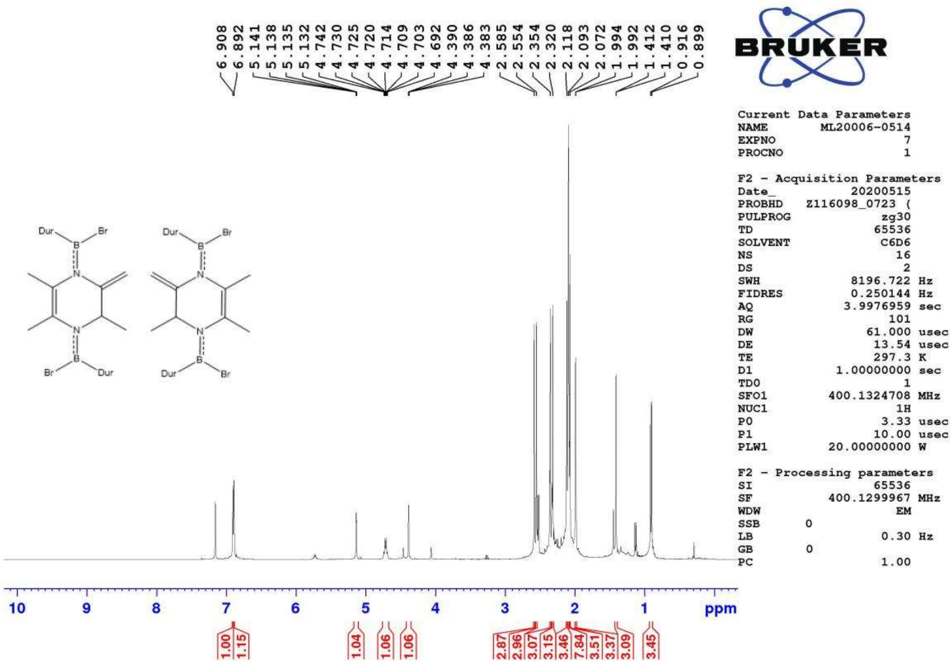

Figure S25. ^{1}H NMR (400 MHz, C_6D_6) of **11a** + **11b**

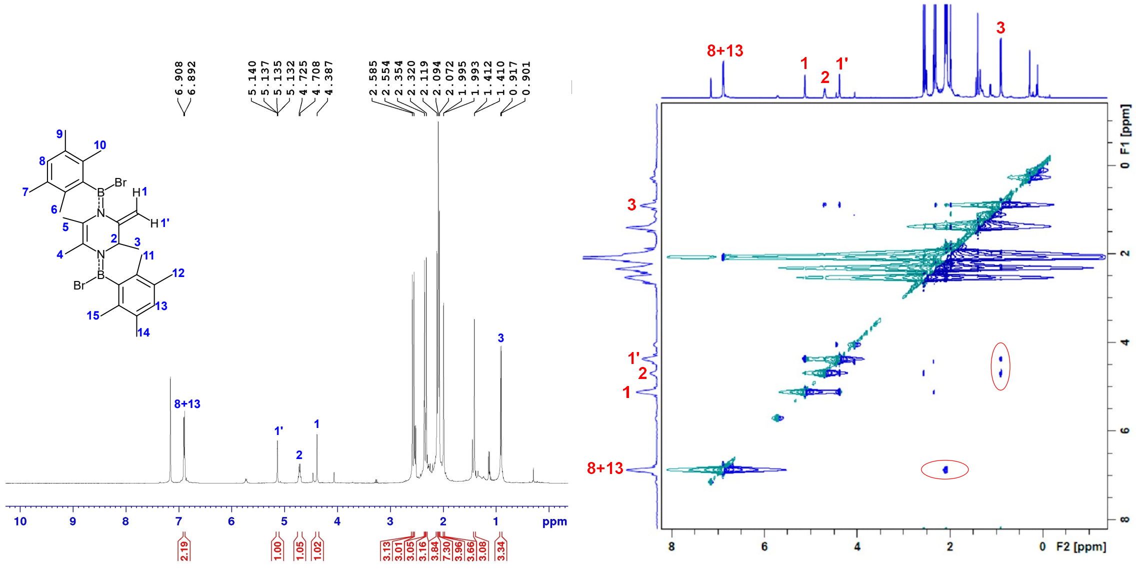

Figure S26. Assignment of **11a**

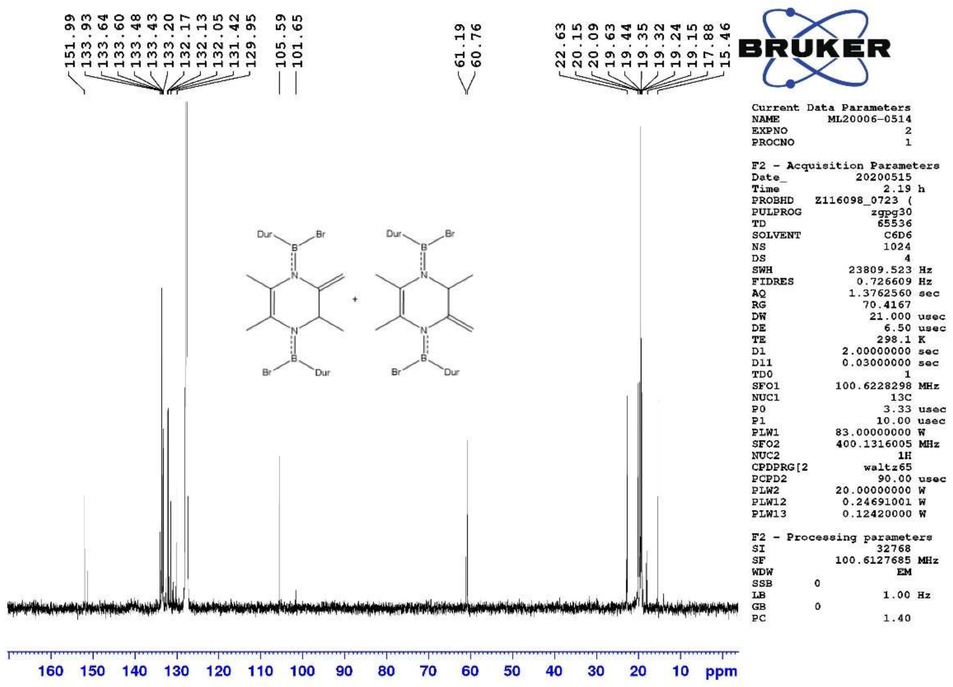

Figure S27. $^{13}C\{^{1}H\}$ NMR (101 MHz, C_6D_6) of **11a** + **11b**

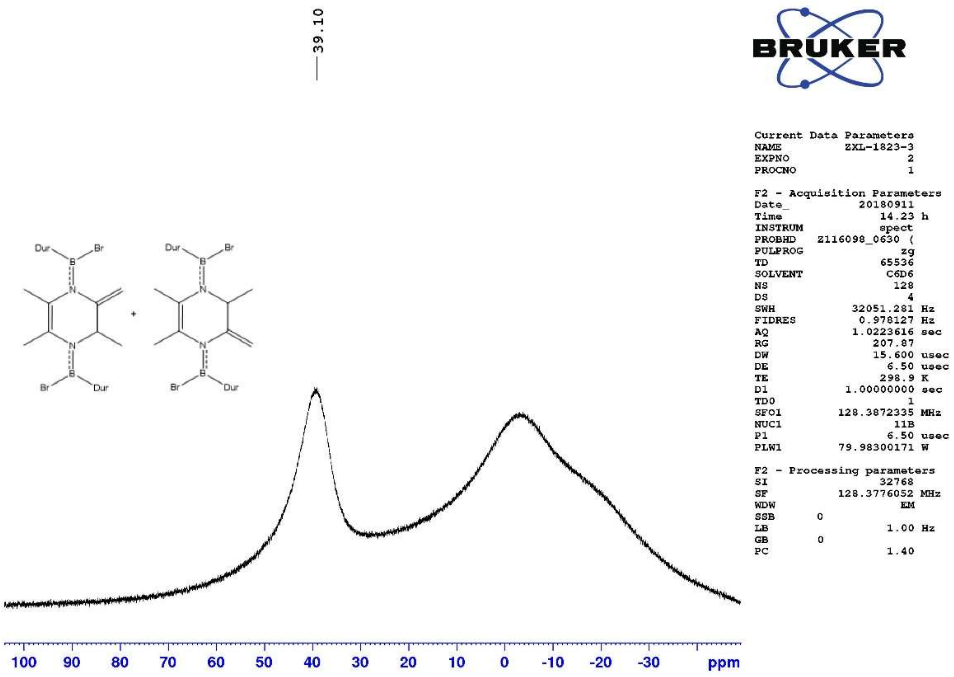

Figure S28. ^{11}B NMR (128 MHz, C_6D_6) of **11a** + **11b**

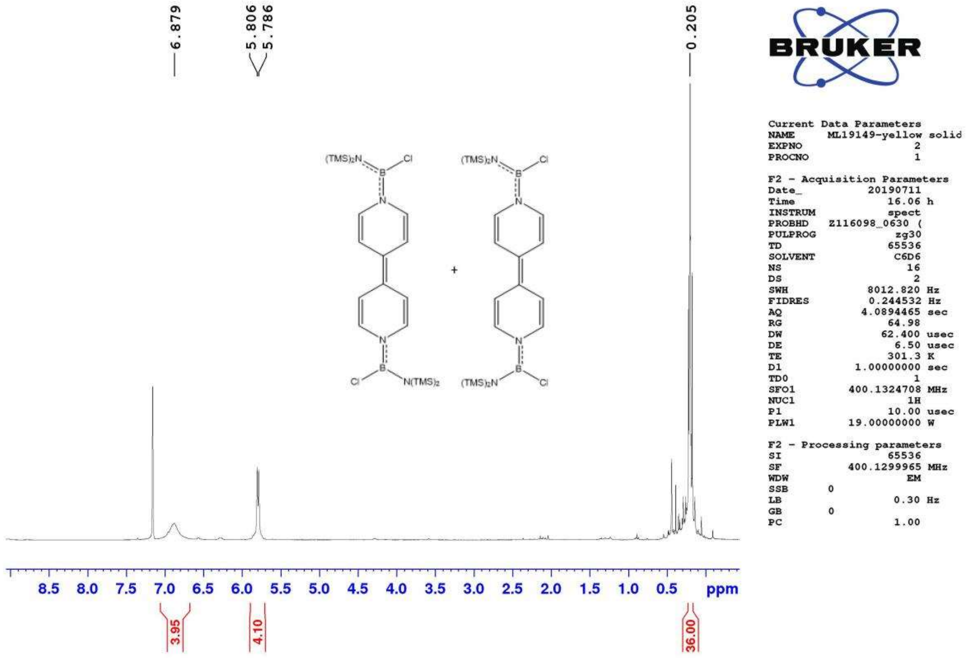

Figure S29. ^{1}H NMR (400 MHz, C_6D_6) of **13a** + **13b**

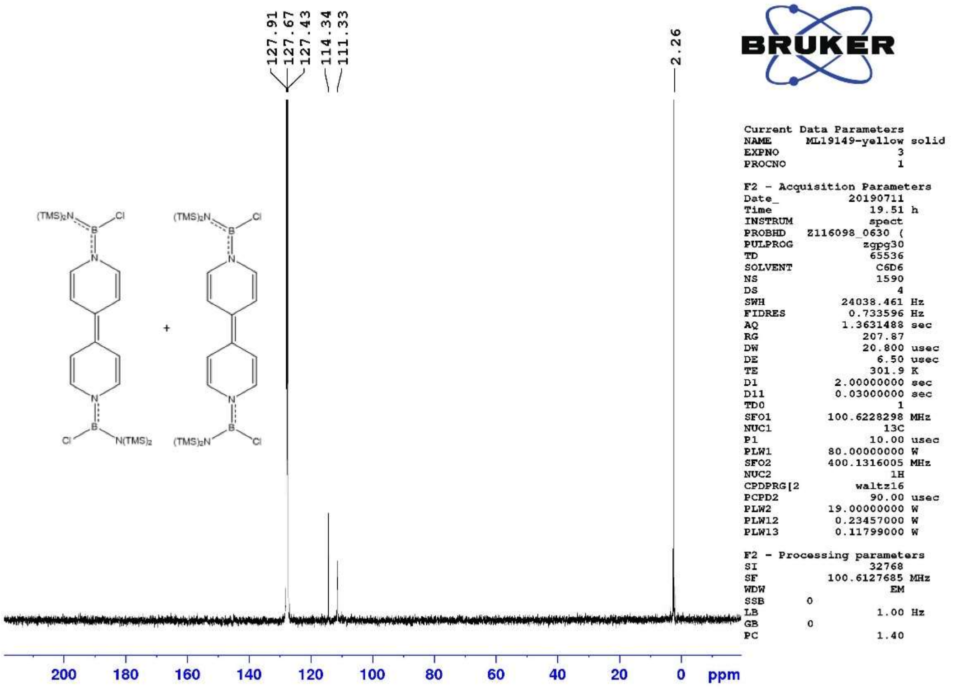

Figure S30. $^{13}C\{^1H\}$ NMR (101 MHz, C_6D_6) of **13a** + **13b**

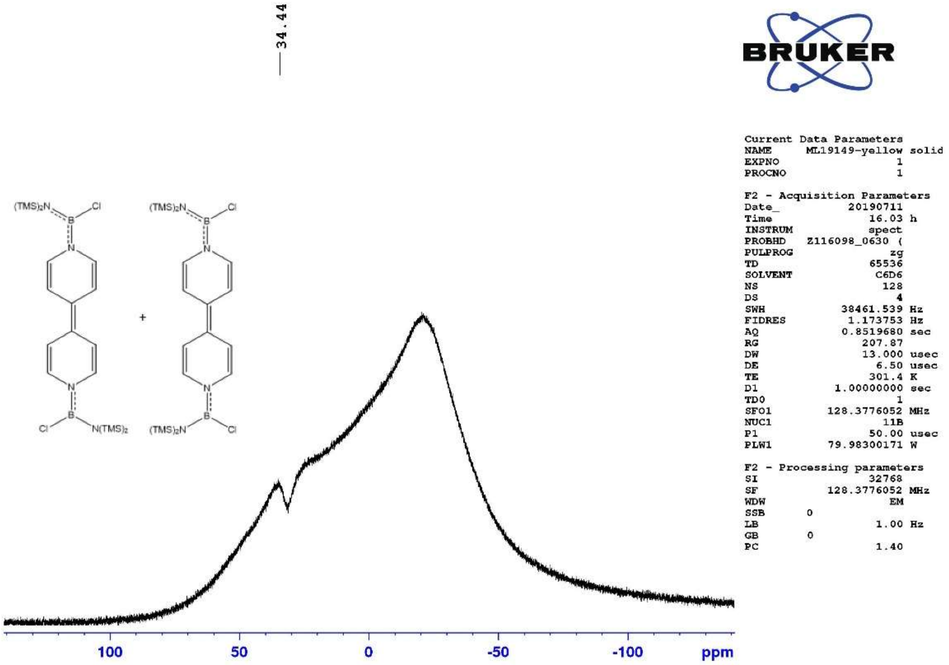

Figure S31. ^{11}B NMR (128 MHz, C_6D_6) of **13a** + **13b**

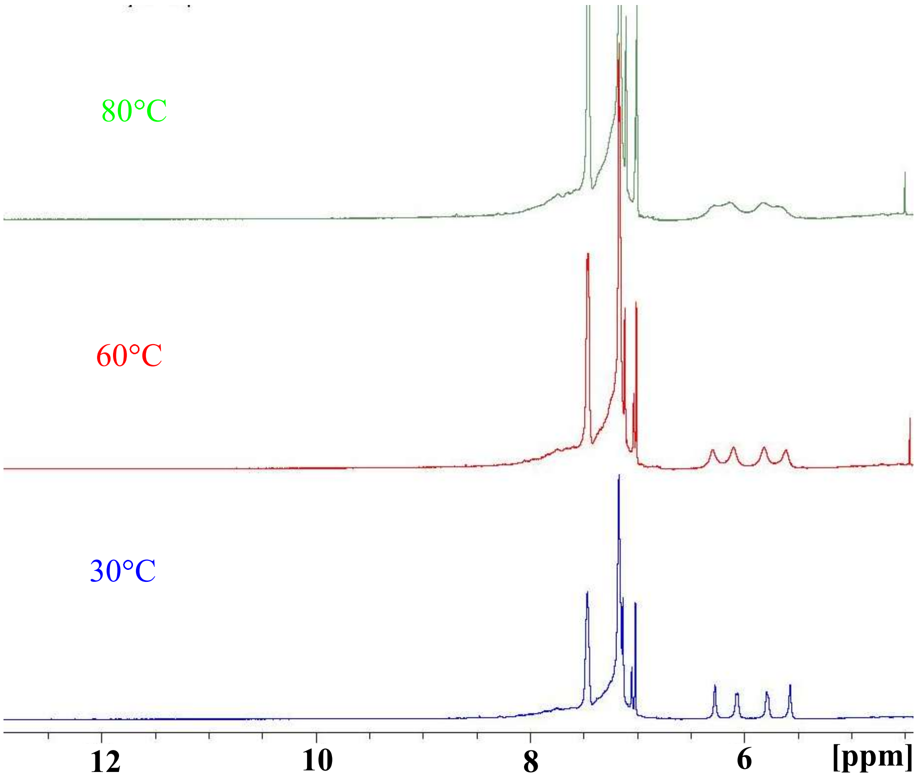

Figure S32. Variable-temperature ^{1}H NMR (400 MHz, toluene-d^8, 30–80 °C) of **7a** + **7b**

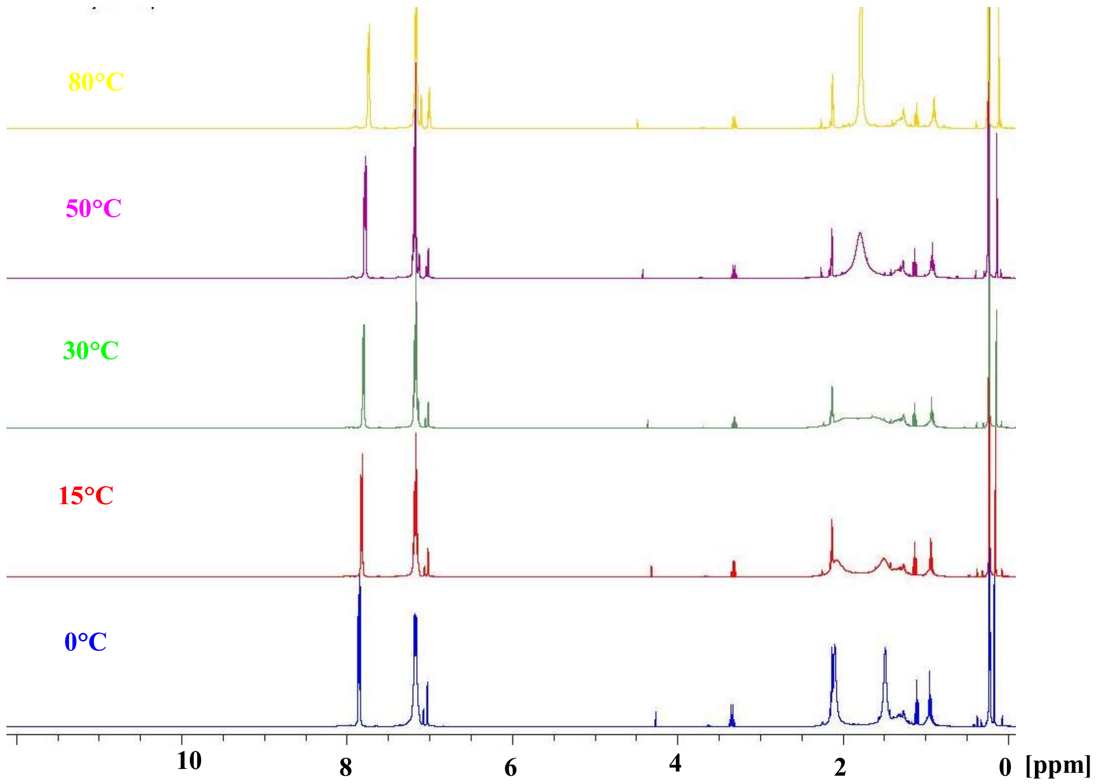

Figure S33. Variable-temperature ^{1}H NMR (400 MHz, toluene-d^8, 0–80 °C) of **10a** + **10b**

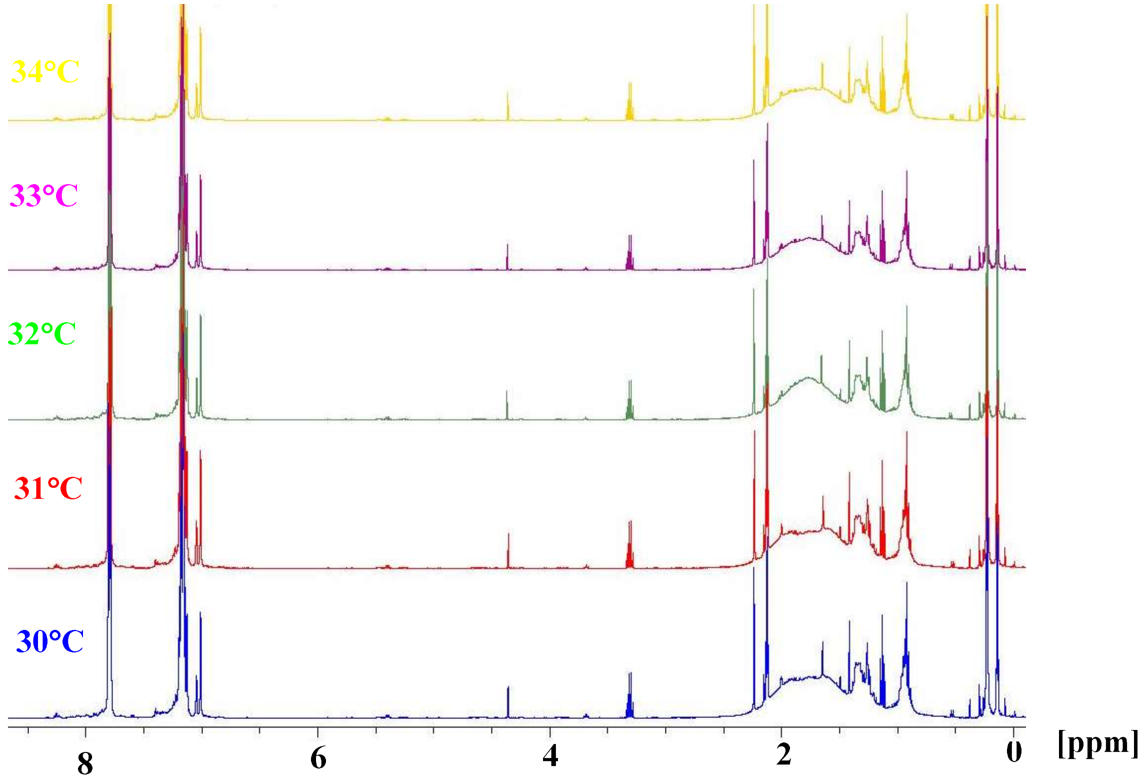

Figure S34. Variable-temperature ^{1}H NMR (400 MHz, toluene-d^8, 30–35 °C) of **10a** + **10b**

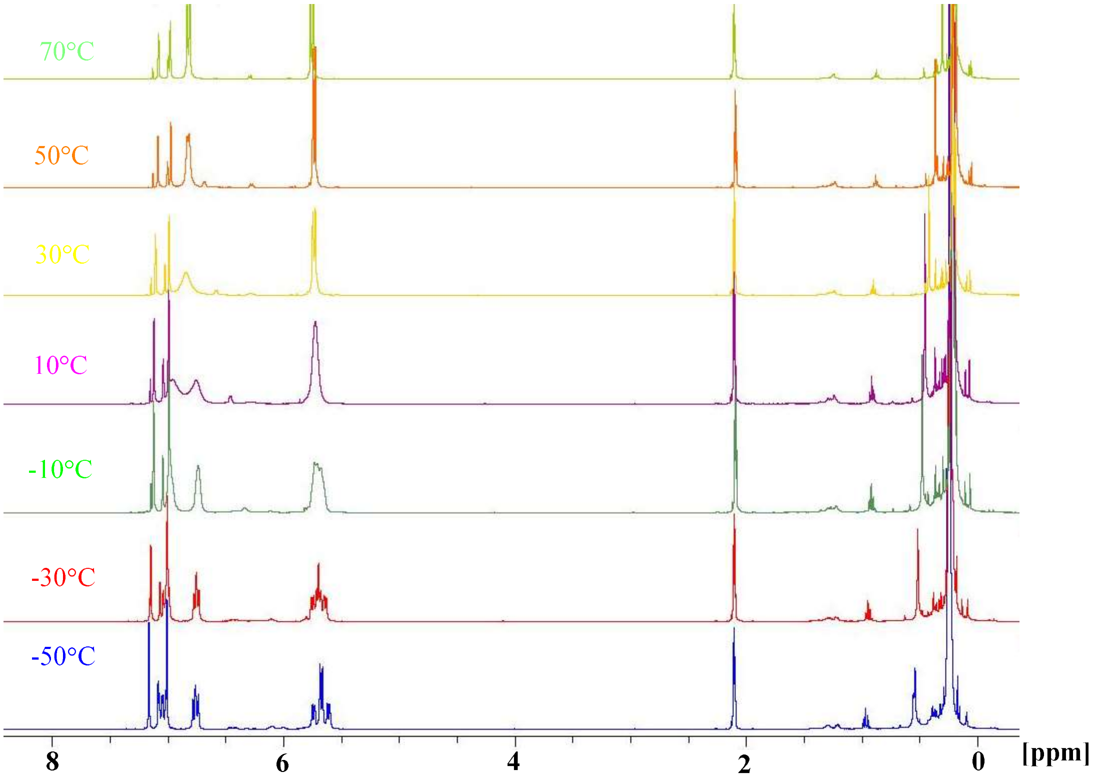

Figure S35. Variable-temperature ^{1}H NMR (400 MHz, toluene-d^{8}, −50–70 °C) of **13a** + **13b**

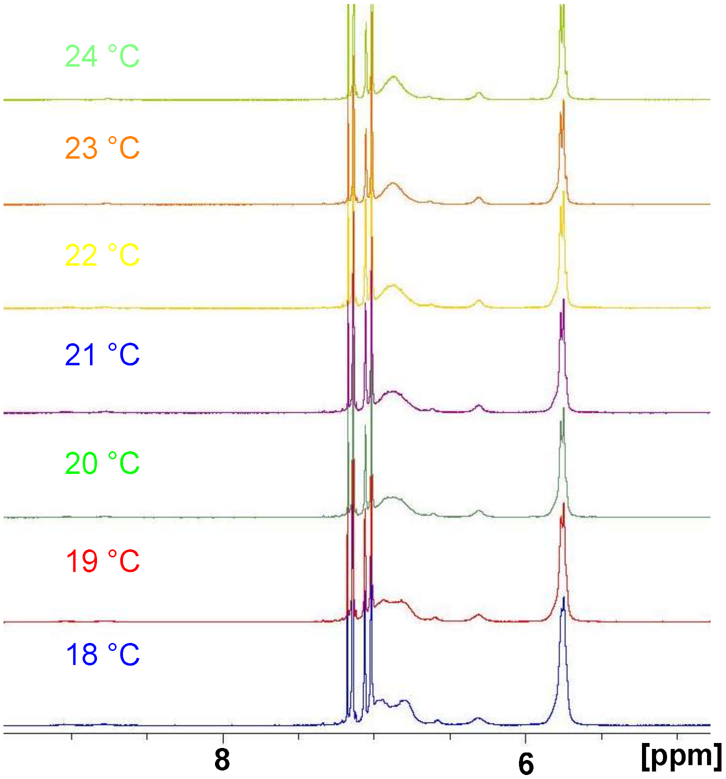

Figure S36. Variable-temperature ^{1}H NMR (400 MHz, toluene-d^{8}, 18–24 °C) of **13a** + **13b**

II. Crystallographic details

X-ray diffraction data for compounds **5a**, **5b**, **9**, **10**, **11a**, **11b** and **13** were collected on a Bruker D8 VENTURE diffractometer with monochromated Mo-Kα radiation (λ = 0.71073 Å). Crystals were kept at 100(2) K throughout collection. Data collection strategy determination, integration, scaling, and space group determination were performed with Apex3 software (Bruker, V2018.1-0). All structures were solved with the ShelXT structure solution program using the Intrinsic Phasing solution method[4] and by using Olex2 as the graphical interface. The model was refined with the ShelXL program[5] using Least Squares minimization. All non-hydrogen atoms were refined anisotropically. Hydrogen atoms were included in structure factor calculations. All hydrogen atoms were assigned to idealized geometric positions. Specific details of each experiment can be found below and in the crystallographic information files.

Crystallographic data have been deposited with the Cambridge Crystallographic Data as supplementary publication nos. CCDC-2006650 (**5**), 2006651 (**9**), 2006652 (**10**), 2006653 (**11**) and 2006654 (**13**). These data can be obtained free of charge from The Cambridge Crystallographic Data Centre *via* www.ccdc.cam.ac.uk/data_request/cif

Table S1. Crystal data for **5**, **9**, **10a**, **11**, and **13a**

Compound	**5a5b**	**9**	**10a**	**11a11b**	**13a**
CCDC number	2006650	2006651	2006652	2006653	2006654
Empirical formula	$C_{24}H_{30}B_2Br_2N_2$	$C_{21}H_{34}BBrN_2Si$	$C_{20}H_{22}B_2Cl_2N_2$	$C_{28}H_{38}B_2Br_2N_2$	$C_{11}H_{22}BClN_2Si_2$
Formula weight	527.94	433.331	382.96	584.04	284.74
Temperature/K	100	100	100	100	100
Crystal system	monoclinic	monoclinic	monoclinic	monoclinic	monoclinic
Space group	C2/c	$P2_1/c$	$P2_1/n$	$P2_1/c$	$P2_1/n$
a/Å	21.8291(16)	18.3958(8)	9.8770(3)	11.8188(9)	6.5493(2)
b/Å	9.2690(5)	8.0538(3)	16.1316(5)	12.6999(10)	31.2087(9)
c/Å	24.5329(18)	17.2745(8)	12.8988(4)	19.1660(15)	8.4426(2)
β/°	92.665(2)	117.711(2)	92.0440(10)	96.897(3)	110.2660(10)
Volume/Å^3	4958.5(6)	2265.78(17)	2053.88(11)	2856.0(4)	1618.80(8)
Z	8	4	4	4	4
ρ_{calc}g/cm^3	1.414	1.270	1.2383	1.358	1.168
μ/mm^{-1}	3.283	1.874	0.322	2.857	0.367
F(000)	2144.0	911.8	801.4	1200.0	608.0
Crystal size/mm^3	0.02 × 0.02 × 0.006	0.4 × 0.2 × 0.2	0.4 × 0.4 × 0.2	0.06 × 0.03 × 0.03	0.123 × 0.122 × 0.111
2Θ range for data collection/°	4.776 to 55.524	4.72 to 55.1	4.84 to 55.08	4.726 to 52.936	5.306 to 54.342
Reflections collected	32598	37496	16251	23838	10394
Independent reflections	5736 [R_{int} = 0.1533, R_{sigma} = 0.1140]	5208 [R_{int} = 0.0435, R_{sigma} = 0.0267]	4698 [R_{int} = 0.0362, R_{sigma} = 0.0350]	5830 [R_{int} = 0.0883, R_{sigma} = 0.0921]	3388 [R_{int} = 0.1578, R_{sigma} = 0.1614]
Data/restraints/parameters	5736/0/279	5208/0/246	4698/0/239	5830/14/340	3388/0/160
Goodness-of-fit on F^2	1.019	1.020	1.052	0.997	1.095
Final R indexes [I>=2σ (I)]	R_1 = 0.0669, wR_2 = 0.1418	R_1 = 0.0631, wR_2 = 0.1685	R_1 = 0.0359, wR_2 = 0.0874	R_1 = 0.0482, wR_2 = 0.0936	R_1 = 0.1345, wR_2 = 0.3141
Final R indexes [all data]	R_1 = 0.1333, wR_2 = 0.1688	R_1 = 0.0677, wR_2 = 0.1707	R_1 = 0.0447, wR_2 = 0.0950	R_1 = 0.1112, wR_2 = 0.1117	R_1 = 0.1889, wR_2 = 0.3617
Largest diff. peak/hole / e Å^{-3}	0.66/-1.03	1.96/-2.31	0.36/-0.31	0.65/-0.98	1.03/-1.55

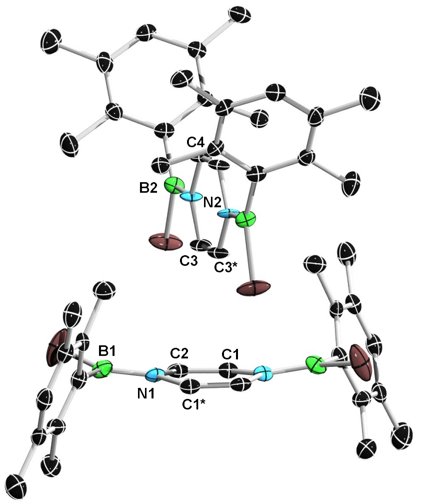

Figure S37. Single crystal structure of **5**. (Hydrogen atoms have been removed for clarity)

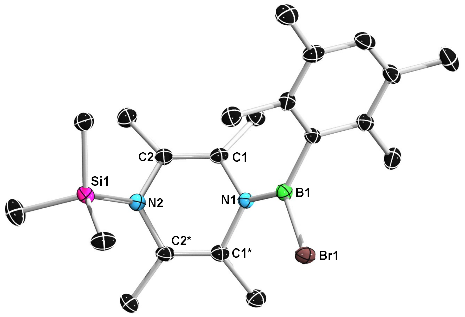

Figure 38. Single crystal structure of **9**. (Hydrogen atoms have been removed for clarity)

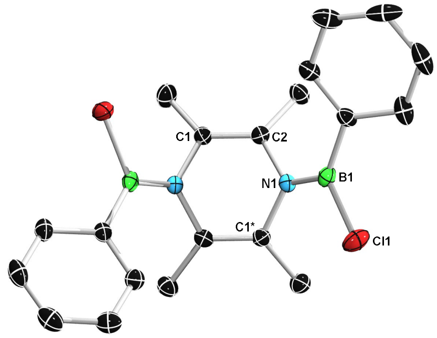

Figure S39. Single crystal structure of **10a**. (Hydrogen atoms have been removed for clarity)

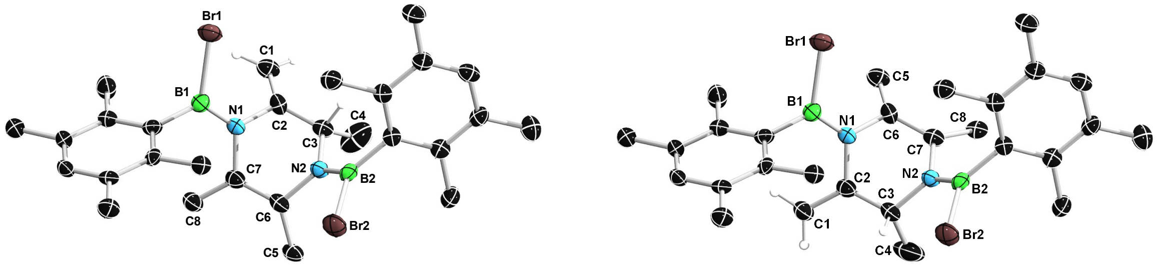

Figure S40. Single crystal structure of **11a** (left) and **11b** (right). (Hydrogen atoms have been removed for clarity)

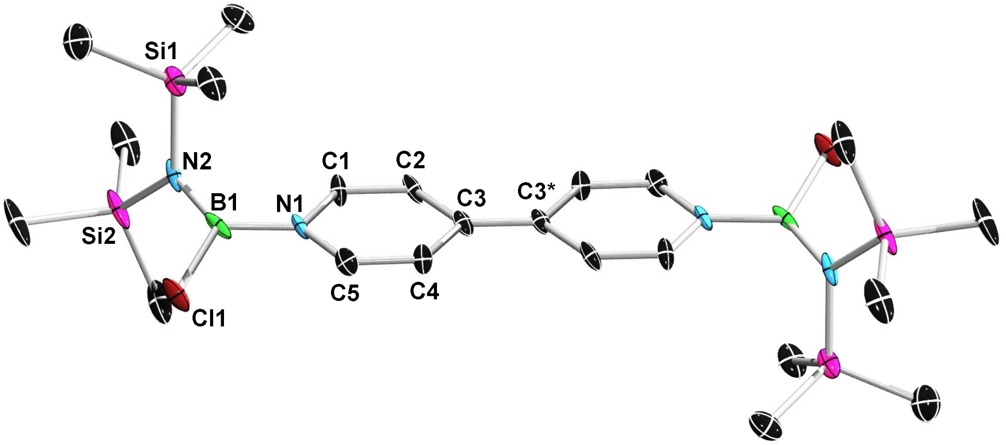

Figure S41. Single crystal structure of **13a**. (Hydrogen atoms have been removed for clarity)
